# Rib fractures and other injuries after cardiopulmonary resuscitation for non-traumatic cardiac arrest: a systematic review and meta-analysis

**DOI:** 10.1007/s00068-023-02421-7

**Published:** 2024-01-11

**Authors:** Suzanne F. M. Van Wijck, Jonne T. H. Prins, Michael H. J. Verhofstad, Mathieu M. E. Wijffels, Esther M. M. Van Lieshout

**Affiliations:** Trauma Research Unit Department of Surgery, Erasmus MC, University Medical Center Rotterdam, P.O. Box 2040, 3000 CA Rotterdam, The Netherlands

**Keywords:** Cardiopulmonary resuscitation, Mechanical CPR, Thoracic injury, Abdominal injury, Surgical stabilization of rib fractures

## Abstract

**Purpose:**

This study aims to ascertain the prevalence of rib fractures and other injuries resulting from CPR and to compare manual with mechanically assisted CPR. An additional aim was to summarize the literature on surgical treatment for rib fractures following CPR.

**Design:**

Systematic review and meta-analysis.

**Data sources:**

Embase, Medline Ovid, Cochrane Central, Web of Science, and Google Scholar.

**Review methods:**

The databases were searched to identify studies reporting on CPR-related injuries in patients who underwent chest compressions for a non-traumatic cardiopulmonary arrest. Subgroup analysis was conducted to compare the prevalence of CPR-related injuries in manual versus mechanically assisted chest compressions. Studies reporting on surgery for CPR-related rib fractures were also reviewed and summarized.

**Results:**

Seventy-four studies reporting CPR-related injuries were included encompassing a total of 16,629 patients. Any CPR-related injury was documented in 60% (95% confidence interval [95% CI] 49–71) patients. Rib fractures emerged as the most common injury, with a pooled prevalence of 55% (95% CI 48–62). Mechanically assisted CPR, when compared to manual CPR, was associated with a higher risk ratio for CPR-related injuries of 1.36 (95% CI 1.17–1.59). Eight studies provided information on surgical stabilization of CPR-related rib fractures. The primary indication for surgery was the inability to wean from mechanical ventilation in the presence of multiple rib fractures.

**Conclusion:**

Rib fractures and other injuries frequently occur in patients who undergo CPR after a non-traumatic cardiopulmonary arrest, especially when mechanical CPR is administered. Surgical stabilization of CPR-related rib fractures remains relatively uncommon.

**Level of evidence:**

Level III, systematic review and meta-analysis.

**Supplementary Information:**

The online version contains supplementary material available at 10.1007/s00068-023-02421-7.

## Introduction

Cardiopulmonary resuscitation (CPR) aims to extend the critical window during which a cardiac arrest’s underlying cause can potentially be reversed by rhythmically applying external force on the anterior chest wall [1]. However, effective CPR comes at a cost.

To compress the chest optimally, the chest wall has to be compressed at least 5 cm in depth [2]. Achieving this requires significant force applied to the chest wall, including the sternum and ribs, as well as adjacent vital structures such as the heart and lungs. Consequently, post-CPR injuries are a common occurrence, although the reported prevalence of these injuries exhibits substantial variability [3]. CPR-related injuries appear to be even more prevalent when mechanical compression devices are employed in conjunction with manual chest compressions [4]. These injuries can range from relatively minor, such as a single undisplaced rib fracture, to life-threatening, such as tension pneumothorax [5]. The wide range in the documented occurrence and severity of injuries following CPR may be attributed to the absence of standardized guidelines for diagnosing and treating CPR-related injuries in post-resuscitation care algorithms [2, 3].

The presence of more than six rib fractures, at least one displaced rib fracture, or a flail chest sustained during CPR is associated with extended hospital length of stay (HLOS) and intensive care unit length of stay (ICU LOS) in survivors of cardiopulmonary arrest [6, 7]. The advantages of surgical stabilization of rib fractures (SSRF) have been increasingly demonstrated, particularly for mechanically ventilated patients with a flail chest due to blunt thoracic trauma. SSRF in this population is associated with reduced pneumonia rates, shorter ICU LOS, and fewer ventilator days [8–11]. However, the evidence regarding the application and benefits of SSRF in patients with CPR-related rib fractures is currently limited [7, 12–18].

The primary objective of this systematic review and meta-analysis was to establish the prevalence of rib fractures and other thoracic and abdominal injuries following CPR for non-traumatic cardiopulmonary arrest, both in cases of manual and mechanically assisted CPR. The secondary objective was to provide an overview of the existing literature on the surgical treatment of rib fractures, which are the most common CPR-related injuries.

## Methods

This study adhered to the Preferred Reporting Items for Systematic Reviews and Meta-Analyses (PRISMA) guideline (Supplementary Online Materials 1) [19]. A protocol was established before this review, but not published. No modifications to the protocol were made during the study’s execution. Approval from the Medical Research Ethics Committee was not deemed necessary.

### Search strategy and selection criteria

The Embase, Medline, Web of Science Core Collection, Cochrane Central Register of Controlled Trials, and Google Scholar were searched on September 12, 2022, for studies pertaining to CPR-related injuries [20]. The search strategies were adapted to accommodate the unique searching features of each database, including database-specific MESH and EMTREE controlled vocabulary terms. Searches were not limited by date, language, or publication status. The search strategy is provided in Supplementary Online Materials 2, which also includes a translated version for use in PubMed. Two reviewers (SFMVW and JTHP) independently screened title and abstract and subsequently reviewed full texts for eligibility. Any disagreements were resolved through consensus. Inclusion criteria encompassed all studies reporting on patients who (a) underwent CPR for non-traumatic cardiac arrest, (b) received chest compressions either manually only or assisted with a mechanical compression device, and (c) underwent autopsy or dedicated imaging enabling identification of CPR-related injuries. Excluded were animal studies, meta-analyses or literature reviews, guidelines or consensus statements, opinion articles, letters to the editor, or conference abstracts. Studies involving pediatric populations or those failing to report any of the CPR-related injuries of interest were also excluded. In cases where a specific population was used more than once in different manuscripts, only the index manuscript was included. Case reports were excluded from the primary prevalence objective but were included in the summary of post-CPR rib fracture management. The reference lists of all included studies were screened to add relevant publications that may have been overlooked in the original search.

### Data extraction

A predefined data sheet was used to extract the data from the included studies. Two reviewers (SFMVW and JTHP) independently performed data extraction and resolved discrepancies through consensus. Extracted data encompassed study characteristics, demographics of the study population, CPR details (such as setting and method), and diagnostic modality for identifying CPR-related injuries.

For the primary objective of this systematic review, collected data were the number of patients and CPR-related injuries. This included the number of rib fractures, their fracture patterns (multiple rib fractures—defined as either two or three or more rib fractures depending on the study, lateral flail chest, and anterior flail segment or flail sternum- defined as three or more bilateral rib fractures in the costochondral or anterior sector of the ribs) [21], characteristics (type of fracture and displacement) [21], and the prevalence of other CPR-related skeletal, soft tissue, cardiac, pulmonary, vascular, and visceral injuries.

For the secondary aim, additional data and outcomes were extracted to summarize the literature about SSRF for CPR-related rib fractures. This included the specific indications, timing, and techniques for SSRF, as well as hospital length of stay, duration of mechanical ventilation, follow-up duration, and mortality.

### Quality assessment and evaluation of publication bias

Two reviewers (SFMVW and JTHP) independently assessed the methodological quality of the included studies using the Methodological index for non-randomized studies (Minors) [22]. Twelve items for studies with a control group and 8 items for studies without a control group were assigned a score of 0 when the item was not reported, 1 when inadequately reported, and 2 when adequately reported (Supplementary Online Materials 3). The total score ranges from 0 (poor quality) to 24 (good quality). Evaluation of publication bias was conducted by visually inspecting funnel plots (Supplementary Online Materials 9–15).

### Statistical analysis

Continuous data are presented as means with standard deviation (SD) or range. Categorical data are expressed as numbers and percentages. Pooled prevalences of CPR-related injuries were calculated using MedCalc (MedCalc Statistical Software version 18.2.1, MedCalc Software bvba, Ostend, Belgium; http://www.medcalc.org; 2018) and reported as percentages with corresponding 95% confidence intervals. Meta-analysis was conducted using ReviewManager (version 5.4, Copenhagen: The Nordic Cochrane Centre, The Cochrane Collaboration, 2020) to compare the prevalence of CPR-related injuries between manual-only and mechanically assisted chest compressions. Heterogeneity was assessed with Cochran’s *Q* test and *I*^2^ statistic. A random effects model was used employed, irrespective of the *Q* test results, due to expected significant heterogeneity. These results are presented as pooled risk ratios with their 95% confidence intervals and *p* value. *p* values < 0.05 were considered statistically significant.

## Results

### Search

**Fig. 1:**
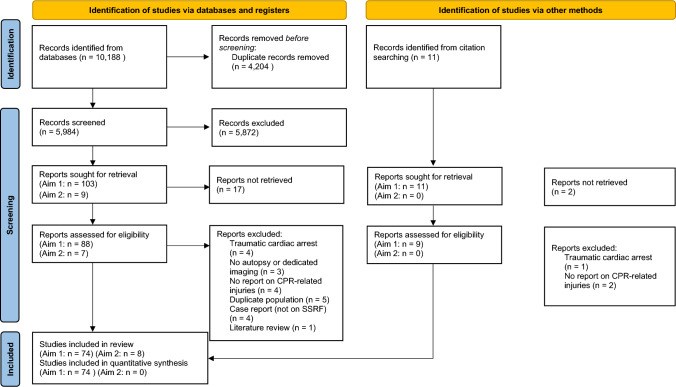
Flow diagram of study selection for aim 1 (prevalence of CPR-related injury) and aim 2 (overview of surgical stabilization for CPR-related rib fractures)

The database search identified 10,188 records and an additional 6 records were included in the meta-analysis through citation searching (Fig. 1). After removing duplicates, titles and abstracts of 6,278 records were screened. The full texts of 104 articles were assessed for eligibility. In total, 74 studies were selected to determine the prevalence of CPR-related injuries. An additional seven studies were selected for the secondary objective to summarize surgical treatment for CPR-related injuries, and one study contributed to both objectives [7, 12–18].

### Study characteristics

The included 74 studies on the prevalence of CPR-related injuries encompassed 16,629 patients (Table 1 and Supplementary Online Materials 4–8). CPR-related injuries were diagnosed through autopsy in 36 studies involving 6,966 (52%) patients [5, 23–57], while CT scans were utilized in 36 studies covering 5,749 (43%) patients [5–7, 23, 34, 41, 43, 58–86]. Of the 74 studies, 45 with 9,931 patients described CPR for out-of-hospital cardiac arrest, while 6 studies with 743 patients reported exclusively on in-hospital cardiac arrest [41, 51, 52, 55, 62, 87]. Furthermore, 29 studies encompassing 2,052 patients detailed injuries following the use of mechanical cardiac compression devices [5, 6, 23–26, 29–32, 37–40, 42, 45, 47, 49–51, 58, 59, 68, 69, 80, 82, 88–90]. Eight other articles involved 57 patients who received surgical treatment for CPR-related rib fractures [7, 12–18].

**Table 1:** Characteristics of studies included in systematic review of CPR-related injuries following non-traumatic cardiac arrest

Author and year	Design	Study period	Diagnostic modality	Total population *N*	Manual CPR *N* (%)	Mechanical CPR *N* (%)	Setting cardiac arrest OHCA *N* (%)	Age mean (SD/P_25_-P_75_/range)	Males *N* (%)
Adel et al*.* (2022) [58]	Retrospective	2018–2021	CT scan	225	NA	NA	225 (100%)	64 (13)	170 (75%)
Azeli et al*.* (2022) [23]	Retrospective	2016	CT scan, radiograph, autopsy	52	0 (0%)	52 (100%)	52 (100%)	57 (49–66)	33 (63%)
Karatasakis et al*.* (2022) [59]	Prospective	2015–2018	CT scan	104	76 (73%)	28 (27%)	104 (100%)	56 (15)	73 (70%)
Katasako et al*.* (2022) [60]	Retrospective	2017–2019	CT scan	306	NA	NA	306 (100%)	81 (71–89)	171 (56%)
Kawai et al*.* (2022) [61]	Retrospective	2015–2019	CT scan	87	NA	NA	87 (100%)	67 (59–75)	55 (63.2%)
Kunz et al*.* (2022) [7]^a^	Retrospective	2018–2019	CT scan, radiograph	109	NA	NA	59 (54%)	69 (56–77)	67 (61%)
Canakci et al*.* (2021) [62]	Retrospective	2015–2020	CT scan	178	131 (74%)	47 (26%)	0 (0%)	73 (65–80)	99 (56%)
Gaisendrees et al*.* (2021) [88]	Retrospective	2016–2020	CT scan, US	108	38 (35%)	70 (65%)	NA	55 (13)	64 (59%)
Hokenek and Erdogan (2021) [63]	Retrospective	2015–2019	CT scan	246	NA	NA	NA	73 (16)	146 (59%)
Karasek et al*.* (2021) [24]	Retrospective	2016–2018	Autopsy	630	559 (90%)	64 (10%)	NA	67	449 (71%)
Prins et al*.* (2021) [6]	Retrospective	2007–2019	CT scan	344	325 (94%(	19 (6%)	344 (100%)	66 (54–74)	259 (75%)
Hwang et al*.* (2021) [64]	Retrospective	2013–2018	CT scan	452	NA	NA	452 (100%)	62 (16)	284 (63%)
Moriguchi et al*.* (2021) [25]	Retrospective	2011–2018	Autopsy	75	65 (87%)	10 (13%)	NA	59 (22)	57 (76%)
Jang et al*.* (2020) [65]	Retrospective	2009–2019	CT scan	43	43 (100%)	0 (0%)	NA	72 (2–98)	27 (37%)
Kim et al*.* (2020) [66]	Retrospective	2007–2016	CT scan	274	274 (100%)	0 (0%)	205 (75%)	63 (15)	180 (66%)
Milling et al*.* (2020) [26]	Prospective	2016–2018	Autopsy	50	0 (0%)	50 (100%)	50 (100%)	48 (38–62)	32 (64%)
Oh and Kim (2020) [67]	Retrospective	2009–2019	CT scan	368	NA	NA	323 (88%)	64	244 (66%)
Sonnemans et al*.* (2020) [68]	Retrospective	2012–2017	Postmortem CT scan	72	29 (40%)	43 (60%)	72 (100%)	59 (47–77)	48 (67%)
Viniol et al*.* (2020) [69]	Retrospective	2016	CT scan	100	93 (93%)	7 (7%)	88 (88%)	69 (13)	73 (73%)
Zaidi et al*.* (2020) [70]	Retrospective	2015–2020	Radiograph, CT scan	137	137 (100%)	0 (0%)	137 (100%)	62 (54–70)	63 (46%)
Zotzmann et al*.* (2020) [71]	Retrospective	2010–2017	CT scan	103	NA	NA	67 (65%)	57 (17)	71 (69%)
Azeli et al*.* (2019) [27]	Prospective	2014–2016	Autopsy	109	109 (100%)	0 (0%)	109 (100%)	63 (49–70)	74 (68%)
Deliliga et al*.* (2019) [28]	Retrospective	2013	Autopsy	88	88 (100%)	0 (0%)	44 (50%)	61 (7.5)	53 (60%)
Friberg et al*.* (2019) [29]	Prospective	2005–2013	Autopsy	414	52 (13%)	362 (87%)	NA	68 (58–77)	284 (69%)
Iglesies et al*.* (2019) [89]	Prospective	2016–2017	Radiograph, CT scan	65	54 (83%)	11 (17%)	65 (100%)	64 (13%)	51 (80%)
Milling et al*.* (2019) [30]	Retrospective	2015–2017	Autopsy, CT scan, US, radiograph, MRI	437	353 (81%)	84 (19%)	437 (100%)	61 (47–73)	322 (74%)
Ondruschka et al*.* (2019) [31]	Retrospective	2017	Autopsy	30	15 (50%)	15 (50%)	NA	59 (15)	30 (100%)
Dunham et al*.* (2018) [72]	Retrospective	2016	CT scan	39	39 (100%)	0 (0%)	39 (100%)	52 (22)	26 (67%)
Ondruschka et al*.* (2018) [32]	Retrospective	2011–2017	Autopsy	614	501 (82%)	113 (18%)	NA	58 (17)	456 (74%)
Setälä et al*.* (2018) [33]	Prospective	2013–2014	Autopsy	149	149 (100%)	0 (0%)	149 (100%)	68 (59–78)	101 (68%)
Takayama et al*.* (2018) [73]	Retrospective	2013–2016	CT scan	472	472 (100%)	0 (0%)	472 (100%)	72 (14)	291 (62%)
Yusufoglu et al*.* (2018) [74]	Retrospective	2014–2016	CT scan	83	NA	NA	NA	67 (12)	48 (58%)
Beom et al*.* (2017) [75]	Retrospective	2006–2015	CT scan	185	185 (100%)	0 (0%)	130 (70%)	63 (18)	110 (59%)
Cha et al*.* (2017) [76]	Retrospective	2006–2010	CT scan	91	NA	NA	91 (100%)	60 (51–74)	49 (54%)
Koster et al*.* (2017) [5]	RCT	2008–2014	Post-mortem CT, autopsy	374	137 (37%)	237 (63%)	156 (42%)	64 (16)	244 (65%)
Nomura et al*.* (2017) [77]	Retrospective	2016–2017	CT scan	100	NA	NA	100 (100%)	71 (2)	45 (45%)
Yamaguchi et al*.* (2017) [34]	Retrospective	2012–2014	Post-mortem CT, autopsy	180	180 (100%)	0 (0%)	154 (86%)	62 (43–73)	119 (66%)
Oya et al*.* (2016) [78]	Retrospective	2010–2012	Radiograph, postmortem CT scan	535	535 (100%)	0 (0%)	535 (100%)	73 (16)	305 (57%)
Ihnát Rudinská et al*.* (2016) [35]	Prospective	2012–2015	Autopsy	80	NA	NA	80 (100%)	58 (5)	61 (76%)
Seung et al*.* (2016) [79]	Retrospective	2009–2014	CT scan	148	NA	NA	89 (60%)	64 (17)	83 (56%)
Vahedian-Azimi et al*.* (2016) [87]	RCT	2014	Radiograph or autopsy	80	80 (100%)	0 (0%)	NA	61 (13)	31 (39%)
Boland et al*.* (2015) [80]	Retrospective	2009–2012	Radiograph, CT scan, MRI, echocardiogram	235	131 (56%)	104 (44%)	235 (100%)	64 (15)	145 (62%)
Kaldırım et al*.* (2015) [36]	Retrospective	2003–2012	Autopsy	203	203 (100%)	0 (0%)	90 (44%)	47 (17)	143 (70%)
Kashiwagi et al*.* (2015) [81]	Retrospective	2008–2013	CT scan postmortem and in survivors	223	223 (100%)	0 (0%)	NA	75 (63–84)	129 (58%)
Koga et al*.* (2015) [82]	Retrospective	2009–2014	Postmortem CT scan	323	82 (25%)	241 (75%)	323 (100%)	78 (66–85)	185 (57%)
Kralj et al*.* (2015) [37]	Retrospective	2004–2013	Autopsy	2,148	2014 (94%)	134 (6%)	1487 (69%)	65 (18–100)	1480 (69%)
Lardi et al*.* (2015) [38]	Retrospective	2011–2013	Autopsy	58	32 (55%)	26 (45%)	NA	53 (18)	38 (66%)
Štěchovský et al*.* (2015) [39]	Retrospective	2012–2013	Autopsy	27	15 (56%)	12 (44%)	3 (11%)	64 (14)	18 (67%)
Choi et al*.* (2014) [83]	Retrospective	2005–2011	CT scan	82	NA	NA	82 (100%)	58 (14–90)	49 (60%)
Smekal et al*.* (2014) [40]	Retrospective	2008–2012	Autopsy	222	83 (37%)	139 (63%)	222 (100%)	67 (21–100)	152 (68%)
Cho et al*.* (2013) [84]	Retrospective	2005–2011	CT scan, radiograph	44	NA	NA	44 (100%)	57 (27–87)	30 (68%)
Hellevuo et al*.* (2013) [41]	Prospective	2009–2011	CT scan, radiograph, autopsy	170	170 (100%)	0 (0%)	0 (0%)	72 (56–80)	110 (65%)
Kim et al*.* (2013) [85]	Prospective	2011	CT scan	71	NA	NA	57 (80%)	65 (55–74)	45 (63%)
Pinto et al*.* (2013) [42]	Retrospective	2005–2009	Autopsy	175	87 (50%)	88 (50%)	NA	51 (15–89)	102 (58%)
Smekal et al*.* (2013) [43]	Retrospective	2008–2011	CT scan, Autopsy	31	NA	NA	31 (100%)	62 (20)	19 (61%)
Charaschaisri et al*.* (2011) [44]	Retrospective	2006–2008	Autopsy	120	NA	NA	NA	40 (13)	60 (79%)
Kim et al*.* (2011) [86]	Retrospective	2009–2010	CT scan, radiograph	40	NA	NA	NA	61 (27–90)	23 (58%)
Smekal et al*.* (2009) [45]	RCT	2005–2007	Autopsy	85	47 (55%)	38 (45%)	71 (84%)	69 (15)	58 (68%)
Meron et al*.* (2007) [90]	Retrospective	1991–2005	Clinical evaluation, US, autopsy	2,558	13 (87%)	2 (13%)	7 (47%)	58 (53–67)	1699 (66%)
Nishida et al*.* (2006) [46]	Prospective		Autopsy of the heart	80	NA	NA	77 (96%)	54 (21)	48 (60%)
Black et al*.* (2004) [47]	Retrospective	2000–2001	Autopsy	499	485 (97%)	14 (3%)	NA	62 (1)	343 (69%)
Lederer et al*.* (2004) [48]	Prospective	1994–2000	Radiograph, autopsy	19	NA	NA	19 (100%)	66 (16)	13 (68%)
Oschatz et al*.* (2001) [91]	Prospective	1997–1999	Radiograph	155	155 (100%)	0 (0%)	NA	58 (51–71)	113 (73%)
Baubin et al*.* (1999) [49]	Prospective		Autopsy	35	20 (57%)	15 (43%)	35 (100%)	61 (23)	25 (71%)
Rabl et al*.* (1996) [50]	Retrospective	1995	Autopsy	56	25 (45%)	31 (55%)	NA	57 (16–86)	44 (78%)
Cohen et al*.* (1993) [51]	RCT	1992–1993	Radiograph, autopsy	62	33 (53%)	29 (47%)	0 (0%)	68 (2)	45 (73%)
Bedell and Fulton (1986) [52]	Retrospective	1981–1983	Autopsy	130	130 (100%)	0 (0%)	0 (0%)	65	82 (63%)
Powner et al*.* (1984) [53]	Retrospective	NA	Autopsy	70	NA	NA	NA	65	50 (72%)
Bjork et al*.* (1982) [92]	Prospective	NA	Clinical evaluation, Radiograph, autopsy	63	63 (100%)	0 (0%)	NA	64	49 (78%)
Murtomaa and Korttila (1974) [93]	Retrospective	1972	Clinical evaluation, autopsy	39	39 (100%)	0 (0%)	39 (100%)	NA	NA
Anthony and Tattersfield (1969) [54]	Retrospective	NA	Autopsy	34	34 (100%)	0 (0%)	NA	NA	NA
Saphir (1968) [55]	Prospective	1966–1697	Autopsy	123	NA	NA	0 (0%)	NA	NA
Lundberg et al*.* (1967) [56]	Retrospective	1964–1966	Autopsy	50	50 (100%)	0 (0%)	NA	NA	NA
Minuck (1966) [57]	Retrospective	1963–1965	Autopsy	63	63 (100%)	0 (0%)	NA	(17–86)	34 (54%)
*SSRF studies*									
DeVoe et al*.* (2022) [12]	Retrospective	2019–2020	NA	5	NA	NA	NA	59 (12)	5 (100%)
Kunz et al*.* (2022) [7]^a^	Retrospective	2018–2019	CT scan, radiograph	4	NA	NA	NA	60 (4)	4 (100%)
Prins et al*.* (2022) [18]	Retrospective	NA	CT scan	39	34 (87%)	5 (13%)	NA	68 (60–73)	34 (87%)
Claydon et al*.* (2020) [13]	Case series	2013–2019	NA	4	4 (100%)	0 (0%)	NA	57 (12)	4 (100%)
Lee et al*.* (2020) [14]	Case report	NA	CT scan	1	1 (100%)	0 (0%)	0 (0%)	57	0 (0%)
Drahos et al*.* (2019) [15]	Case report	NA	CT scan	1	1 (100%)	0 (0%)	1 (100%)	59	1 (100%)
Pouwels et al*.* (2018) [16]	Case series	NA	CT scan	2	0 (0%)	2 (100%)	2 (100%)	71 (8)	2 (100%)
Ananiadou et al*.* (2010) [17]	Case report	NA	Physical examination	1	1 (100%)	0 (0%)	0 (0%)	59	1 (100%)

*CT* computed tomography, *NA* not available, *OHCA* out-of-hospital cardiac arrest, *RCT* randomized clinical trial, *SSRF* surgical stabilization of rib fractures, *US* ultrasound

^a^This study is mentioned twice because it provided data for both objectives

### Quality assessment and evaluation of publication bias

The methodological quality assessment is presented in Supplementary Online Materials 3. The mean score across all included studies was 13 points (range 6–23). For the 33 studies with a control group, the mean score was 17 points (range 11–23) [5–7, 23, 24, 29–32, 38–40, 42, 44, 45, 49–51, 58–63, 68, 74, 75, 78, 79, 82, 87, 88, 91]. For the 41 studies without a control group, the mean score was 10 points (range 6–13) [25–28, 33–37, 41, 43, 46–48, 52–56, 64–67, 69–73, 76, 77, 80, 81, 83–86, 89, 90, 92–94]. The mean score for the studies addressing SSRF for post-CPR rib fractures was 10 points (range 7–18) [7,12–18]. Visual inspection of the funnel plots did not raise concerns regarding substantial publication bias (Supplementary Online Materials 9–13) [16, 17, 20, 21].

### CPR-related injuries

The prevalence of any CPR-related injury was reported in 35 studies, involving 7,208 patients (Table 2 and Supplementary Online Materials 4–8) [6, 24–26, 31, 32, 35–38, 40, 41, 45, 48–53, 55, 57, 59, 60, 63, 65, 69, 70, 72, 73, 77, 80, 88, 89, 92, 93]. The pooled prevalence of any CPR-related injury was 60% (95% confidence interval [95% CI] 49–71). The most frequent skeletal injury was one or more rib fractures, with a pooled prevalence of 55% (95% CI 48–62) from 60 studies, totaling 12,110 patients [5–7, 23–25, 27–38, 40, 41, 43–45, 47–56, 58–63, 65–67, 69, 72–81, 83–87, 89, 93]. An anterior flail segment was described in five studies, with a pooled prevalence 36% (95% CI 22–50) in 923 patients [6, 7, 29, 59, 89]. The pooled prevalence of sternum fractures was 24% (95% CI 18–30) from 61 studies, encompassing 12,061 patients [5–7, 23–38, 40–45, 47–50, 52–55, 58–63, 65–69, 72–76, 79–87, 89, 93, 94]. The most common pulmonary injury was pulmonary contusion, with a pooled prevalence of 20% (95% CI 12–29) from 29 studies, involving 5,070 patients [5, 6, 24–26, 30, 33–36, 38, 41, 52, 59, 62, 63, 65–67, 69, 74–76, 79, 80, 83–85, 89]. A retrosternal hematoma was the most prevalent cardiac injury, with a pooled prevalence of 12% (95% CI 7.3–18) from 13 studies, covering 2,599 patients [27, 29, 40, 43, 45, 60, 65–67, 75, 76, 79, 82]. The highest prevalence of CPR-related abdominal injury was liver injury, with 3% (95% CI 12.1–4.5) from 27 studies, involving 9,369 patients [5, 24–27, 29, 30, 32–34, 36–38, 40–43, 45, 58, 68, 72, 80, 89, 90, 92–94]. Based on six studies with 905 patients, the prevalence of other abdominal injuries was 4% (95% CI 1.3–7.8), including blunt abdominal trauma without further specification, mesenteric injury, and retroperitoneal hemorrhage [34, 42, 64, 71, 82, 89].

**Table 2:** Pooled prevalence of CPR-related injuries

	Studies	Population	Cases	Heterogeneity		Pooled prevalence (%)
	*N*	*N*	*N*	Cochran’s *Q* (*p* value)	*I*^2^ (%) (95% CI)	(95% CI)
Any CPR-related injury	35	7,208	4,574	2,766 (< 0.001)	99 (99–99)	60.2 (49.3–70.7)
Thoracic wall injury						
Sternum fracture	61	12,061	3,813	4024 (< 0.001)	99 (98–99)	23.6 (17.5–30.2)
Upper third	10	225	33	31 (< 0.001)	72 (46–85)	11.0 (4.31–20.3)
Middle third	10	225	159	68 (< 0.001)	87 (78–92)	74.2 (56.5–88.6)
Lower third	10	225	74	95 (< 0.001)	91 (85–94)	23.0 (7.61–43.5)
Flail sternum	5	923	398	68 (< 0.001)	94 (89–97)	35.6 (22.2–50.3)
Rib fracture	60	12,110	7,294	3435 (< 0.001)	98 (98–98)	55.2 (48.2–62.0)
Bilateral rib fractures	19	2,420	1,093	751 (< 0.001)	98 (97–98)	37.1 (25.1–50.0)
Multiple rib fractures	26	3,952	1,977	1381 (< 0.001)	98 (98–98)	50.3 (38.6–61.9)
Flail chest	7	1,231	38	20 (0.003)	70 (35–86)	3.85 (2.00–6.30)
Clavicle fracture	2	305	1	0 (0.682)	0 (0–0)	0.54 (0.03–1.67)
Scapula fracture	4	662	9	2 (0.506)	0 (0–83)	1.52 (0.73–2.59)
Vertebral fracture	15	3,795	36	48 (< 0.001)	71 (51–83)	1.17 (0.59–1.93)
Extrathoracic chest wall injury	15	3,360	361	433 (< 0.001)	97 (96–98)	8.01 (3.58–14.01)
Pneumomediastinum	8	1,149	33	17 (0.020)	58 (8–81)	3.22 (1.68–5.22)
Hemomediastinum	22	4,068	175	115 (< 0.001)	82 (73–88)	4.80 (3.31–6.55)
Pulmonary injury						
Hemothorax	36	6,886	615	1007 (< 0.001)	97 (96–97)	10.1 (6.53–14.3)
Pneumothorax	43	8,038	545	320 (< 0.001)	87 (83–90)	7.03 (5.49–8.74)
Tension pneumothorax	4	833	9	3 (0.462)	0 (0–85)	1.23 (0.59–2.08)
Pulmonary contusion	29	5,070	1,020	1601 (< 0.001)	98 (98–99)	20.2 (12.4–29.3)
Pulmonary hematoma	6	1,123	36	41 (< 0.001)	88 (76–94)	3.28 (0.89–7.10)
Pulmonary laceration	7	1,357	17	32 (< 0.001)	81 (62–91)	2.18 (0.65–4.58)
Bone marrow or fat embolism	5	333	33	34 (< 0.001)	88 (75–94)	11.5 (3.36–23.7)
Other pulmonary injury	3	823	117	167 (< 0.001)	99 (98–99)	29.4 (3.01–68.1)
Cardiac injury						
Cardiac contusion	10	1,725	66	70 (< 0.001)	87 (78–92)	6.43 (3.23–10.6)
Cardiac laceration, rupture, perforation	13	2,954	50	27 (0.007)	56 (18–76)	1.98 (1.24–2.90)
Pericardial or epicardial injury	28	5,490	282	305 (< 0.001)	91 (88–93)	5.72 (3.76–8.05)
Retrosternal hematoma	13	2,599	304	190 (< 0.001)	94 (91–96)	11.9 (7.30–17.5)
Other cardiac injury	11	2,234	105	90 (< 0.001)	89 (82–93)	4.62 (2.20–7.88)
Abdominal injury						
Stomach injury	9	3,515	16	44 (< 0.001)	82 (66–90)	1.42 (0.45–2.92)
Liver injury	27	9,369	183	219 (< 0.001)	88 (84–91)	3.15 (2.07–4.46)
Spleen injury	18	5,066	44	86 (< 0.001)	80 (69–87)	1.40 (0.66–2.40)
Pancreas injury	3	901	3	2 (0.302)	16 (0–97)	0.44 (0.08–1.10)
Kidney injury	5	1,031	11	16 (0.003)	75 (39–90)	2.01 (0.49–4.52)
Bowel injury	3	797	4	0 (0.918)	0 (0–61)	0.67 (0.22–1.35)
Hemoperitoneum	12	2,963	101	184 (< 0.001)	94 (91–96)	3.79 (1.37–7.34)
Pneumoperitoneum	9	1,871	27	17 (0.028)	54 (2–78)	1.65 (0.89–2.65)
Other abdominal injury	6	905	36	29 (< 0.001)	83 (64–92)	3.87 (1.25–7.83)
Vascular injury						
Thoracic vascular injury	22	5,664	57	85 (< 0.001)	75 (63–84)	1.83 (1.09–2.76)
Abdominal aorta injury	3	338	3	2 (0.318)	13 (0–97)	1.21 (0.25–2.87)
Other injury						
Trachea injury	2	544	2	1 (0.355)	0 (0–0)	0.50 (0.08–1.26)
Diaphragm injury	2	664	2	3 (0.096)	64 (0–92)	0.82 (0.05–4.10)

*CI* confidence interval, *CPR* cardiopulmonary resuscitation

### Manual only versus mechanically assisted CPR

Twenty studies compared CPR-related injury prevalence between manual-only and mechanically assisted CPR, encompassing a total of 2,336 patients in the manual and 1,716 patients in the mechanical group [5, 23, 24, 29–32, 38–40, 42, 45, 49, 50, 59, 62, 68, 82, 88, 89]. Overall, mechanical CPR was associated with a higher risk for all reported injuries. The risk ratio (RR) for any CPR-related injury was higher (1.36 (95% CI 1.17–1.59)) for the patients receiving CPR with mechanical compressions than for those receiving only manual compressions (Table 3 and Supplementary Online Materials 14, 15, 18, 19). Mechanical CPR was also associated with a higher risk of rib fractures with a RR 1.27 (95% CI 1.11–1.45). Other injuries with a higher risk associated with mechanical CPR included myocardial contusion (RR 8.71, 95% CI 3.02–25.1) and bowel injury (RR 7.93, 95% CI 1.12–56.3). Specifically, for piston type mechanical CPR devices, the risk ratio was higher for thoracic injuries, including sternum fractures (RR 1.81, 95% CI 1.30–2.53), flail chest (RR 4.29, 95% CI 1.23–14.99), hemothorax (RR 4.20, 95% CI 2.04–8.68), and especially myocardial contusion (RR 19.79, 95% CI 2.58–151.46). CPR with mechanical load distributing band devices was associated with a higher risk for pneumothorax (RR 2.61, 95% CI 1.00–6.78).

**Table 3:** Comparison between the prevalence of CPR-related injuries between manual and mechanical compressions, and subdivided by mechanical device

Outcome	Manual vs mechanical				Manual vs piston				Manual vs LDB			
	Studies	Manual	Mechanical	RR	Studies	Manual	Piston	RR	Studies	Manual	LDB	RR
Any CPR-related injury	11	654/1450 (45%)	439/550 (80%)	1.36 (1.17–1.59)	8	504/761 (66%)	387/447 (87%)	1.32 (1.13–1.55)	–	–	–	–
Thoracic wall injury												
Sternum fracture	17	884/2272 (39%)	640/1556 (41%)	1.49 (1.14–1.95)	10	262/1259(21%)	514/870 (59%)	1.81 (1.30–2.53)	3	61/198 (31%)	58/372 (16%)	0.68 (0.25–1.84)
Flail sternum	2	43/128 (34%)	199/390 (51%)	1.43 (0.38–5.32)	–	–	–	–	–	–	–	–
Rib fracture	14	1160/2074 (56%)	861/1184 (73%)	1.27 (1.11–1.45)	10	539/1259(43%)	663/870 (76%)	1.30 (1.12–1.51)	–	–	–	–
Multiple rib fractures	4	147/535 (27%)	464/623 (74%)	1.46 (1.13–1.88)	4	147/535 (27%)	464/623 (74%)	1.46 (1.13–1.88)	–	–	–	–
Flail chest	2	12/405 (3,0%)	195/446 (44%)	4.29 (1.23–14.99)	2	12/405 (3,0%)	195/446 (44%)	4.29 (1.23–14.99)	–	–	–	–
Vertebral fracture	5	3/1612 (0,2%)	10/531 (1,9%)	4.73 (1.31–17.1)	2	0/937 (0,0%)	3/336 (0,9%)	5.89 (0.66–52.92)	–	–	–	–
Extrathoracic chest wall injury	4	131/984 (13%)	83/374 (22%)	9.44 (0.73–122.45)	4	131/984 (13%)	83/374 (22%)	9.44 (0.73–122.45)	–	–	–	–
Hemomediastinum	5	26/759 (3,4%)	44/680 (6,5%)	1.89 (1.16–3.08)	4	21/683 (3,1%)	39/652 (6,0%)	1.87 (0.96–3.64)	–	–	–	–
Pulmonary injury												
Hemothorax	9	42/1916 (2,2%)	62/1304 (4,8%)	1.79 (0.91–3.55)	5	11/1120 (1,0%)	25/745 (3,4%)	4.20 (2.04–8.68)	2	18/111 (16%)	30/284 (11%)	0.84 (0.52–1.36)
Pneumothorax	11	40/1518 (2,6%)	80/1376 (5,8%)	1.86 (1.23–2.82)	7	26/1205 (2,2%)	22/853 (2,6%)	1.45 (0.57–3.71)	2	7/111 (6,3%)	46/284 (16%)	2.61 (1.00–6.78)
Pulmonary contusion	6	111/1277(8,7%)	50/460 (11%)	1.56 (0.67–3.61)	3	65/516 (13%)	37/157 (24%)	1.47 (0.29–7.44)	–	–	–	–
Pulmonary hematoma	4	2/220 (0,9%)	12/609 (2,0%)	2.12 (0.62–7.29)	4	2/220 (0,9%)	12/609 (2,0%)	1.92 (0.52–7.12)	–	–	–	–
Pulmonary laceration	2	2/429 (0,5%)	2/112 (1,8%)	3.05 (0.58–16.02)	–	–	–	–	–	–	–	–
Other pulmonary injury	2	45/530 (8,5%)	51/156 (33%)	1.91 (0.25–14.51)	–	–	–	–	–	–	–	–
Cardiac injury												
Cardiac contusion	3	7/927 (0,8%)	11/160 (6,9%)	8.71 (3.02–25.10)	2	0/368 (0,0%)	8/96 (8,3%)	19.79 (2.58–151.46)	–	–	–	–
Cardiac laceration, rupture, perforation	4	10/1137 (0,9%)	13/570 (2,3%)	4.53 (1.92–10.70)	3	2/578 (0,3%)	11/506 (2,2%)	5.14 (0.90–29.44)	–	–	–	–
Pericardial or epicardial injury	11	74/1852 (4,0%)	97/1208 (8,0%)	1.81 (1.32–2.48)	7	49/1106 (4,4%)	62/832 (7,5%)	1.91 (1.29–2.82)	2	11/111 (10%)	32/284 (11%)	1.43 (0.79–2.61)
Retrosternal hematoma	4	37/264 (14%)	117/780 (15%)	1.36 (0.98–1.89)	3	21/182 (12%)	62/539 (12%)	1.49 (0.96–2.32)	-	–	–	–
Other cardiac injury	5	37/1242 (3,0%)	15/716 (2,1%)	1.49 (0.77–2.89)	4	12/683 (1,8%)	12/652 (1,8%)	1.70 (0.73–3.99)	–	–	–	–
Abdominal injury												
Stomach injury	2	0/405 (0,0%)	2/446 (0,4%)	2.34 (0.09–62.85)	2	0/405 (0,0%)	2/446 (0,4%)	2.34 (0.09–62.85)	–	–	–	–
Liver injury	10	41/1869 (2,2%)	67/1168 (5,7%)	2.05 (0.84–5.03)	6	14/1068 (1,3%)	55/762 (7,2%)	3.91 (1.97–7.73)	2	12/116 (10%)	8/131 (6,1%)	0.46 (0.21–1.01)
Spleen injury	5	19/1075 (1,8%)	10/317 (3,2%)	1.63 (0.40–6.59)	2	1/400 (0,3%)	3/122 (2,5%)	6.24 (0.93–42.08)	2	9/116 (7,8%)	5/131 (3,8%)	0.57 (0.07–4.40)
Pancreas injury	2	0/405 (0,0%)	2/446 (0,4%)	2.34 (0.09–62.85)	2	0/405 (0,0%)	2/446 (0,4%)	2.34 (0.09–62.85)	–	–	–	–
Kidney injury	4	3/466 (0,6%)	12/515 (2,3%)	1.99 (0.69–5.71)	3	1/437 (0,2%)	10/472 (2,1%)	3.46 (0.66–18.06)	–	–	–	–
Bowel injury	2	0/440 (0,0%)	3/172 (1,7%)	7.93 (1.12–56.28)	–	–	–		–	–	–	–
Hemoperitoneum	5	16/1533 (1,0%)	68/572 (11,9%)	2.88 (1.64–5.07)	3	9/892 (1,0%)	28/267 (10%)	2.71 (1.35–5.46)	–	–	–	–
Pneumoperitoneum	3	1/237 (0,4%)	10/495 (2,0%)	2.56 (0.54–12.20)	–	–	–	–	2	1/111 (0,9%)	9/284 (3,2%)	2.32 (0.38–14.25)
Other abdominal injury	2	1/169 (0,6%)	19/329 (5,8%)	5.33 (0.96–29.48)	–	–	–	–	2	1/169 (0,6%)	19/329 (5,8%)	4.99 (0.92–27.13)
Vascular injury												
Thoracic vascular injury	6	7/753 (0,9%)	21/748 (2,8%)	2.86 (1.14–7.16)	6	7/753 (0,9%)	21/748 (2,8%)	0.02 (0.01–0.04)	–	–	–	–
Abdominal aorta injury	2	0/130 (0,0%)	2/177 (1,1%)	2.59 (0.28–23.70)	2	0/130 (0,0%)	2/177 (1,1%)	2.58 (0.27–24.49)	–	–	–	–

*CI* confidence interval, *CPR* cardiopulmonary resuscitation

### Surgical management of CPR-related rib fractures

Despite pooling data from over 12,000 patients with CPR-related rib fractures, only eight studies reported on a total of 57 patients who underwent SSRF for these fractures [7, 12–18]. The largest study comprised 39 patients [18]. The pooled mean age was 65 (SD 10) years and, and a total of 51 patients (89%) were male. The most frequent indication for SSRF was the inability to wean patients with multiple rib fractures off mechanical ventilation, with a specific pattern being a flail sternum in the majority (93%) of patients. The time interval between CPR and SSRF ranged from 1 to 38 days. Several fixation systems were employed, including pectus bars, sternal fixation plates, and rib fixation systems including MatrixRIB™ (Synthes), RibFixBlu™ (Zimmer Biomet), and RibLoc® U + (Acute innovations). Postoperatively, 18 cases (41%) of pneumonia and one case (2%) of surgical site infection were reported. One study documented postoperative thoracic bleeding, occurring in six patients (15%). Successful weaning from mechanical ventilation was reported in 17 (94%) patients. The majority of patients (n = 44, 83%) were discharged alive.

## Discussion

The primary objective of this systematic review and meta-analysis was to determine the prevalence of rib fractures and other thoracic and abdominal injuries associated with CPR for non-traumatic cardiopulmonary arrest. Two-thirds of the patients sustained CPR-related injuries, with rib fractures being the most common (55%). Additionally, over one-third of patients had a flail sternum following CPR. Notably, CPR-related injuries, including rib fractures and cardiac injuries, were more frequently identified after mechanically assisted CPR than after manual CPR alone. In particular, piston type mechanical CPR devices were associated with more injuries compared to load distributing band devices and manual-only CPR. In addition, surgical stabilization of CPR-related rib fractures is infrequently performed and the number of reports on surgical stabilization of CPR-related rib fractures was too limited to conduct a formal meta-analysis.

The prevalence of CPR-related injuries in this review exceeded the 32–45% reported in a systematic review published in 2014 [4]. Several factors may explain this increased prevalence, including the improved imaging by CT scans instead of plain radiographs and the utilization and development of mechanical devices to assist CPR. Other contributing factors to the increased prevalence and its range may include the quality of chest compressions, the setting in which CPR was administered, and the characteristics of the studied population.

The current review highlighted that CPR assisted with a mechanical device resulted in more injuries compared to manual compressions alone, consistent with previous literature [95]. For instance, prior studies have reported a two- to tenfold increase in the prevalence of rib and sternum fractures due to mechanically assisted CPR, with the extent of the increase varying depending on the type of mechanical device employed [4]. Nevertheless, the higher prevalence of cardiac injuries following mechanical CPR has not been previously described, which may be attributed to changes in resuscitation guidelines over the years. These guidelines currently recommend deeper chest compressions, potentially resulting in more injuries [96]. Furthermore, the inclusion of CT scans for diagnosing certain causes of cardiac arrest may have contributed to the increased identification of CPR related injuries, since CT is a significantly more sensitive modality for detecting CPR-related injuries than physical examination or radiographs alone [86]. Additionally, CT has also proven to be a valuable adjunct to autopsy for diagnosing CPR-related injuries [43].

To date, no literature reviews have been published on SSRF of CPR-related rib fractures, with most available publications being limited to case reports or case series involving a maximum of five patients [12]. Therefore, quantitative synthesis of these results was deemed not useful due to the selection of the cardiac arrest patients with the best anticipated outcome, coupled with a low number of patients in these studies. Nonetheless, the summarized findings suggest that SSRF of CPR-related rib fractures may lead to favorable respiratory outcomes in post-CPR patients with an anterior flail chest who fail to be weaned from mechanical ventilation. Important to note is that the number of post-CPR SSRF cases was low and surgical timing and technique were heterogeneous. Additionally, the current literature on SSRF generally does not address patients with CPR-related rib fractures, as these patients are typically excluded from clinical trials on the subject. Consequently, future comparative studies, preferably prospective ones, are required to provide guidance on the optimal management of CPR survivors with severe rib fracture patterns.

Several limitations should be acknowledged in this systematic review and meta-analysis. First, selection bias may have been present, as patients who underwent autopsy or diagnostic imaging following CPR could differ systematically from those who did not. Second, not all studies provided information the CPR setting, and some study populations included both out-of-hospital and in-hospital cardiac arrest cases. Additionally, the background of the CPR provider was not consistently reported, making it impossible to distinguish between chest compressions administered by healthcare providers, bystanders, or a combination of both. Third, the CPR duration, an important risk factor for CPR-related injuries, could not be consistently accounted for in the meta-analysis due to incomplete or inconsistent reporting of data [30, 32, 65, 77, 79, 81, 84]. Moreover, CPR-guidelines have evolved since their first description in 1960, potentially impacting the prevalence and patterns of CPR-related injuries over the past six decades, as deeper compressions are likely to result in more injuries [1, 2, 74, 75, 78]. Last, this meta-analysis included CPR-related injuries detected using different diagnostic modalities, including physical examination, radiographs, CT-scans conducted after return of spontaneous circulation, post-mortem CT, autopsy, or a combination of these modalities. Factors such as the study period, diagnostic modality, and survivor status may potentially have influenced the prevalence of injuries identified.

With the improved sensitivity of the diagnostic modalities in recent years, the true prevalence of CPR-related injury is becoming clearer. The added value of studies reporting on CPR-related injuries diagnosed solely with radiographs and without the current state-of-the-art diagnostic modalities is questionable. A meta-analysis that exclusively includes studies combining physical exams with high-resolution CT for survivors and/or autopsy for non-survivors would reveal a higher, yet more accurate CPR-related injury prevalence. The subsequent question would revolve around the optimal treatment for these CPR-related injuries, particularly in the case of CPR-related rib fractures, where the question arises of when and if to perform SSRF.

In conclusion, CPR-related thoracic injuries are frequently identified in post-CPR patients following a non-traumatic cardiopulmonary arrest. These injuries can be serious and consequential. Mechanically assisted CPR is associated with a higher risk of CPR-related compared to manual compressions alone. Surgical stabilization of CPR-related rib fractures is currently performed incidentally.

## Supplementary Information

Below is the link to the electronic supplementary material.

Supplementary file1 (PDF 6241 KB)

## Data Availability

Upon request mailed to the corresponding author.
