# Supplementary material for: Rib fractures and other injuries after cardiopulmonary resuscitation for non-traumatic cardiac arrest: a systematic review and meta-analysis
Source: Eur J Trauma Emerg Surg. 2024 Jan 11;50(4):1331–46. doi: 10.1007/s00068-023-02421-7 (PMC11458643; doi:10.1007/s00068-023-02421-7)
Supplement: Supplementary file 1 — Supplementary file1 (PDF 6241 KB) [file 68_2023_2421_MOESM1_ESM.pdf]

SUPPLEMENTAL ONLINE MATERIALS

To article: Rib fractures and other injuries after cardiopulmonary resuscitation for non-traumatic cardiac arrest: a systematic review and meta-analysis

European Journal of Trauma and Emergency Surgery

Authors: Suzanne F.M. Van Wijck, MD, Jonne T.H. Prins, MD PhD, Michael H.J. Verhofstad, MD PhD, Mathieu M.E Wijffels, MD PhD, Esther M.M. Van Lieshout, PhD MSc

Trauma Research Unit Department of Surgery Erasmus MC, University Medical Center Rotterdam

Corresponding author e-mail address: e.vanlieshout@erasmusmc.nl

1. Completed Preferred Reporting Items for Systematic reviews and Meta-Analyses (PRISMA) checklist
2. Literature search strategy for the systematic review of CPR-related injuries following non-traumatic cardiac arrest
3. Quality assessment following the MINORS criteria of studies included in systematic review of the prevalence of CPR-related injuries following non-traumatic cardiac arrest
4. Forest plots for any CPR-related injury and thoracic injuries
5. Forest plots for CPR-related pulmonary injuries
6. Forest plots for CPR-related cardiac injuries
7. Forest plots for CPR-related abdominal injuries
8. Forest plots for other CPR-related injuries

9. Funnel plots for any CPR-related injury and thoracic injuries
10. Funnel plots for CPR-related pulmonary injuries
11. Funnel plots for CPR-related cardiac injuries
12. Funnel plots for CPR-related abdominal injuries
13. Funnel plots for other CPR-related injuries
14. Forest plots detailing the risk ratio for CPR-related thoracic injury of manual versus mechanical CPR following non-traumatic cardiac arrest
15. Forest plots detailing the risk ratio for CPR-related abdominal injury of mechanical versus manual CPR following non-traumatic cardiac arrest
16. Funnel plots for any or thoracic CPR-related injuries sustained during manual versus mechanically assisted CPR
17. Funnel plots for abdominal CPR-related injuryies sustained during manual versus mechanically assisted CPR
18. Forest plots detailing the risk ratio for CPR-related thoracic injury of manual versus mechanical CPR with a piston device following non-traumatic cardiac arrest
19. Forest plots detailing the risk ratio for CPR-related thoracic injury of manual versus mechanical CPR with a load distributing band device following non-traumatic cardiac arrest
20. Funnel plots for CPR-related injuries sustained during manual versus mechanically assisted CPR with a piston device
21. Funnel plots for CPR-related injuries sustained during manual versus mechanically assisted CPR with a load distributing band devic

Supplementary Online Materials 1: Completed Preferred Reporting Items for Systematic reviews and Meta-Analyses (PRISMA) checklist

Section and Topic	Item #	Checklist item	Location where item is reported
**TITLE**			
Title	1	Identify the report as a systematic review.	Title
**ABSTRACT**			
Abstract	2	See the PRISMA 2020 for Abstracts checklist.	Abstract
**INTRODUCTION**			
Rationale	3	Describe the rationale for the review in the context of existing knowledge.	Page 1-2 of introduction
Objectives	4	Provide an explicit statement of the objective(s) or question(s) the review addresses.	Page 2 of introduction
**METHODS**			
Eligibility criteria	5	Specify the inclusion and exclusion criteria for the review and how studies were grouped for the syntheses.	Page 1-2 of methods
Information sources	6	Specify all databases, registers, websites, organizations, reference lists and other sources searched or consulted to identify studies. Specify the date when each source was last searched or consulted.	Page 1 of methods
Search strategy	7	Present the full search strategies for all databases, registers and websites, including any filters and limits used.	Supplementary Online Materials 2
Selection process	8	Specify the methods used to decide whether a study met the inclusion criteria of the review, including how many reviewers screened each record and each report retrieved, whether they worked independently, and if applicable, details of automation tools used in the process.	Page 1 of methods
Data collection process	9	Specify the methods used to collect data from reports, including how many reviewers collected data from each report, whether they worked independently, any processes for obtaining or confirming data from study investigators, and if applicable, details of automation tools used in the process.	Page 1-2 of methods
Data items	10a	List and define all outcomes for which data were sought. Specify whether all results that were compatible with each outcome domain in each study were sought (e.g. for all measures, time points, analyses), and if not, the methods used to decide which results to collect.	Page 2 of methods
	10b	List and define all other variables for which data were sought (e.g. participant and intervention characteristics, funding sources). Describe any assumptions made about any missing or unclear information.	Page 2 of methods
Study risk of bias assessment	11	Specify the methods used to assess risk of bias in the included studies, including details of the tool(s) used, how many reviewers assessed each study and whether they worked independently, and if applicable, details of automation tools used in the process.	Page 2-3 of methods, Supplementary

Section and Topic	Item #	Checklist item	Location where item is reported
			Online Materials 3
Effect measures	12	Specify for each outcome the effect measure(s) (e.g. risk ratio, mean difference) used in the synthesis or presentation of results.	Page 3 of methods
Synthesis methods	13a	Describe the processes used to decide which studies were eligible for each synthesis (e.g. tabulating the study intervention characteristics and comparing against the planned groups for each synthesis (item #5)).	Page 2-3 of methods
	13b	Describe any methods required to prepare the data for presentation or synthesis, such as handling of missing summary statistics, or data conversions.	Page 2-3 of methods
	13c	Describe any methods used to tabulate or visually display results of individual studies and syntheses.	Page 2 of methods
	13d	Describe any methods used to synthesize results and provide a rationale for the choice(s). If meta-analysis was performed, describe the model(s), method(s) to identify the presence and extent of statistical heterogeneity, and software package(s) used.	Page 3 of methods
	13e	Describe any methods used to explore possible causes of heterogeneity among study results (e.g. subgroup analysis, meta-regression).	Page 3 of methods
	13f	Describe any sensitivity analyses conducted to assess robustness of the synthesized results.	n/a
Reporting bias assessment	14	Describe any methods used to assess risk of bias due to missing results in a synthesis (arising from reporting biases).	Page 2-3 of methods
Certainty assessment	15	Describe any methods used to assess certainty (or confidence) in the body of evidence for an outcome.	Page 2-3 of methods
**RESULTS**			
Study selection	16a	Describe the results of the search and selection process, from the number of records identified in the search to the number of studies included in the review, ideally using a flow diagram.	Page 1 of results, figure 1
	16b	Cite studies that might appear to meet the inclusion criteria, but which were excluded, and explain why they were excluded.	n/a
Study characteristics	17	Cite each included study and present its characteristics.	Page 1 of results , table 1
Risk of bias in studies	18	Present assessments of risk of bias for each included study.	Page 1-2 of results, Supplementary Online Materials 3
Results of individual studies	19	For all outcomes, present, for each study: (a) summary statistics for each group (where appropriate) and (b) an effect estimate and its precision (e.g. confidence/credible interval), ideally using structured tables or plots.	Page 2-4 of results

Section and Topic	Item #	Checklist item	Location where item is reported
Results of syntheses	20a	For each synthesis, briefly summarize the characteristics and risk of bias among contributing studies.	Supplementary Online Materials 9-13, 16-17, 20-21
	20b	Present results of all statistical syntheses conducted. If meta-analysis was done, present for each the summary estimate and its precision (e.g. confidence/credible interval) and measures of statistical heterogeneity. If comparing groups, describe the direction of the effect.	Page 2-4 of results, table 2
	20c	Present results of all investigations of possible causes of heterogeneity among study results.	Page 3&4 of results
	20d	Present results of all sensitivity analyses conducted to assess the robustness of the synthesized results.	n/a
Reporting biases	21	Present assessments of risk of bias due to missing results (arising from reporting biases) for each synthesis assessed.	n/a
Certainty of evidence	22	Present assessments of certainty (or confidence) in the body of evidence for each outcome assessed.	Page 2-4 of results
**DISCUSSION**			
Discussion	23a	Provide a general interpretation of the results in the context of other evidence.	Page 1-3 of discussion
	23b	Discuss any limitations of the evidence included in the review.	Page 2&3 of discussion
	23c	Discuss any limitations of the review processes used.	Page 3 of discussion
	23d	Discuss implications of the results for practice, policy, and future research.	Page 3 of discussion
**OTHER INFORMATION**			
Registration and protocol	24a	Provide registration information for the review, including register name and registration number, or state that the review was not registered.	Page 1 of methods
	24b	Indicate where the review protocol can be accessed, or state that a protocol was not prepared.	Page 1 of methods
	24c	Describe and explain any amendments to information provided at registration or in the protocol.	n/a
Support	25	Describe sources of financial or non-financial support for the review, and the role of the funders or sponsors in the review.	Title page
Competing interests	26	Declare any competing interests of review authors.	Statement at end of manuscript

Section and Topic	Item #	Checklist item	Location where item is reported
Availability of data, code and other materials	27	Report which of the following are publicly available and where they can be found: template data collection forms; data extracted from included studies; data used for all analyses; analytic code; any other materials used in the review.	Statement at end of manuscript

Supplementary Online Materials 2: Literature search strategy for the systematic review of CPR-related injuries following non-traumatic cardiac arrest

Embase (4637 records, 4580 after removal of duplicates)

('resuscitation'/de OR 'mechanical chest compressor'/de OR 'heart massage'/de OR 'manual compression'/de OR (resuscit* OR reanimat* OR CPR OR ((chest) NEAR/3 (compression*)) OR ((basic OR advanced-cardiac*) NEXT/2 (life) NEXT/2 (support*)) OR BLS OR ((active* OR automated) NEAR/3 (compression*)) OR ((mechanical) NEAR/2 (chest) NEAR/2 (compress*)) OR LUCAS OR Autopulse OR ((heart* OR cardiac*) NEAR/3 (massage)) OR (manual NEAR/3 compression*)):ab,ti,kw) AND ('heart arrest'/exp OR 'heart ventricle fibrillation'/de OR 'heart arrhythmia'/de OR 'cardiopulmonary arrest'/de OR (((heart* OR cardiac* OR cardio* OR circulat*) NEAR/6 (arrest* OR standstill* OR stand-still*)) OR asystole* OR asystolia* OR OHCA OR IHCA OR ((ventric*) NEAR/3 (fibrillat*)) OR arrhythm*):ab,ti,kw) AND ('thorax injury'/de OR 'lung contusion'/de OR 'pneumothorax'/de OR 'hematopneumothorax'/de OR 'tension pneumothorax'/de OR 'hematothorax'/de OR 'rib fracture'/exp OR 'sternal fracture'/de OR 'sternum fracture'/de OR 'heart tamponade'/de OR 'bleeding'/de OR 'respiratory tract hemorrhage'/de OR 'pneumomediastinum'/de OR 'heart injury'/de OR 'commotio cordis'/de OR 'heart muscle injury'/de OR 'blood vessel injury'/de OR 'aortic trauma'/de OR 'artery injury'/de OR 'vein injury'/de OR 'mechanical chest compressor'/'adverse device effect' OR (((thorax* OR thoracic* OR heart* OR cardiac* OR cardial* OR lung* OR pulmonar* OR chest* OR cor OR myocard* OR blood-vessel* OR vein* OR aorta* OR aortic OR artery OR arteries OR arterial OR vascular) NEAR/3 (injur* OR trauma* OR damage*)) OR ((pulmonar* OR lung* OR cor OR myocard* OR thorax* OR thoracic* OR heart OR cardiac) NEAR/3 (contusion* OR bruis* OR concussion*)) OR pneumothorax* OR hematopneumothorax* OR haematopneumothorax OR haemopneumothorax OR hemopneumothorax OR hematothorax OR haematothorax OR haemothorax OR hemathorax OR haemathorax OR hemothorax OR ((flail) NEAR/3 (chest* OR thorax OR thoracic* OR sternum*)) OR ((rib* OR costa* OR sternum OR sternal) NEAR/3 (fractur* OR damage* OR injur* OR trauma*)) OR ((heart OR cardiac* OR cardial* OR pericardi* OR percardi*) NEAR/3 (tamponad*)) OR bleeding OR haemorrhage* OR hemorrhage* OR ((blood) NEAR/3 (loss)) OR pneumomediastinum* OR ((mediastin*) NEAR/3 (emphysem*)) OR commotio-cordis):ab,ti,kw) NOT ((animal/exp OR animal*:de OR nonhuman/de) NOT ('human'/exp)) NOT ([Conference Abstract]/lim)

Medline (2706 records, 631 after removal of duplicates)

(Resuscitation/ OR exp Cardiopulmonary Resuscitation/ OR Heart Massage/ OR (resuscit* OR reanimat* OR CPR OR ((chest) ADJ3 (compression*)) OR ((basic OR advanced-cardiac*) ADJ2 (life) ADJ2 (support*)) OR BLS OR ((active* OR automated) ADJ3 (compression*)) OR ((mechanical) ADJ2 (chest) ADJ2 (compress*)) OR LUCAS OR Autopulse OR ((heart* OR cardiac*) ADJ3 (massage)) OR (manual ADJ3 compression*)).ab,ti,kf.) AND (exp Heart Arrest/ OR Ventricular Fibrillation/ OR Arrhythmias, Cardiac/ OR (((heart* OR cardiac* OR cardio* OR circulat*) ADJ6 (arrest* OR standstill* OR stand-still*)) OR asystole* OR asystolia* OR OHCA OR IHCA OR ((ventric*) ADJ3 (fibrillat*)) OR arrhythm*).ab,ti,kf.) AND (exp Thoracic Injuries/ OR Pneumothorax/ OR exp Hemothorax/ OR Rib Fractures/ OR Cardiac Tamponade/ OR Hemorrhage/ OR Mediastinal Emphysema/ OR Vascular System Injuries/ OR Resuscitation/ae OR Heart Massage/ae OR (((thorax* OR thoracic* OR heart* OR cardiac* OR cardial* OR lung* OR pulmonar* OR chest* OR cor OR myocard* OR blood-vessel* OR vein* OR aorta* OR aortic OR artery OR arteries OR arterial OR vascular) ADJ3 (injur* OR trauma* OR damage*)) OR ((pulmonar* OR lung* OR cor OR myocard* OR thorax* OR thoracic* OR heart OR cardiac) ADJ3 (contusion* OR bruis* OR concussion*)) OR pneumothorax* OR hematopneumothorax* OR haematopneumothorax OR haemopneumothorax OR hemopneumothorax OR hematothorax OR haematothorax OR haemothorax OR hemathorax OR haemathorax OR hemothorax OR ((flail) ADJ3 (chest* OR thorax OR thoracic* OR sternum*)) OR ((rib* OR costa* OR sternum OR sternal) ADJ3 (fractur* OR damage* OR injur* OR trauma*)) OR ((heart OR cardiac* OR cardial* OR pericardi* OR percardi*) ADJ3 (tamponad*)) OR bleeding OR haemorrhage* OR hemorrhage* OR ((blood) ADJ3 (loss)) OR pneumomediastinum* OR ((mediastin*) ADJ3 (emphysem*)) OR commotio-cordis).ab,ti,kf.) NOT (exp animals/ NOT humans/) NOT (news OR congres* OR abstract* OR book* OR chapter* OR dissertation abstract*).pt.

Cochrane (156 records, 39 after removal of duplicates)

((resuscit* OR reanimat* OR CPR OR ((chest) NEAR/3 (compression*)) OR ((basic OR advanced NEXT cardiac*) NEXT/2 (life) NEXT/2 (support*)) OR BLS OR ((active* OR automated) NEAR/3 (compression*)) OR ((mechanical) NEAR/2 (chest) NEAR/2 (compress*)) OR LUCAS OR Autopulse OR ((heart* OR cardiac*) NEAR/3 (massage)) OR (manual NEAR/3 compression*)):ab,ti,kw) AND ((((heart* OR cardiac* OR cardio* OR circulat*) NEAR/6 (arrest* OR standstill* OR stand NEXT still*)) OR asystole* OR asystolia* OR OHCA OR IHCA OR ((ventric*) NEAR/3 (fibrillat*)) OR arrhythm*):ab,ti,kw) AND ((((thorax* OR thoracic* OR heart* OR cardiac* OR cardial* OR lung* OR pulmonar* OR chest* OR cor OR myocard* OR blood NEXT vessel* OR vein* OR aorta* OR aortic OR artery OR arteries OR arterial OR vascular) NEAR/3 (injur* OR trauma* OR damage*)) OR ((pulmonar* OR lung* OR cor OR myocard* OR thorax* OR thoracic* OR heart OR cardiac) NEAR/3 (contusion* OR bruis* OR concussion*)) OR pneumothorax* OR hematopneumothorax* OR haematopneumothorax OR haemopneumothorax OR hemopneumothorax OR hematothorax OR haematothorax OR

haemothorax OR hemathorax OR haemathorax OR hemothorax OR ((flail) NEAR/3 (chest* OR thorax OR thoracic* OR sternum*)) OR ((rib* OR costa* OR sternum OR sternal) NEAR/3 (fractur* OR damage* OR injur* OR trauma*)) OR ((heart OR cardiac* OR cardial* OR pericardi* OR percardi*) NEAR/3 (tamponad*)) OR bleeding OR haemorrhage* OR hemorrhage* OR ((blood) NEAR/3 (loss)) OR pneumomediastinum* OR ((mediastin*) NEAR/3 (emphysem*)) OR commotio NEXT cordis):ab,ti,kw)

Web of Science (2395 records, 626 after removal of duplicates)

TS=(((resuscit* OR reanimat* OR CPR OR ((chest) NEAR/2 (compression*)) OR ((basic OR advanced-cardiac*) NEAR/2 (life) NEAR/2 (support*)) OR BLS OR ((active* OR automated) NEAR/2 (compression*)) OR ((mechanical) NEAR/2 (chest) NEAR/2 (compress*)) OR LUCAS OR Autopulse OR ((heart* OR cardiac*) NEAR/2 (massage)) OR (manual NEAR/2 compression*))) AND ((((heart* OR cardiac* OR cardio* OR circulat*) NEAR/5 (arrest* OR standstill* OR stand-still*)) OR asystole* OR asystolia* OR OHCA OR IHCA OR ((ventric*) NEAR/2 (fibrillat*)) OR arrhythm*)) AND ((((thorax* OR thoracic* OR heart* OR cardiac* OR cardial* OR lung* OR pulmonar* OR chest* OR cor OR myocard* OR blood-vessel* OR vein* OR aorta* OR aortic OR artery OR arteries OR arterial OR vascular) NEAR/2 (injur* OR trauma* OR damage*)) OR ((pulmonar* OR lung* OR cor OR myocard* OR thorax* OR thoracic* OR heart OR cardiac) NEAR/2 (contusion* OR bruis* OR concussion*)) OR pneumothorax* OR hematopneumothorax* OR haematopneumothorax OR haemopneumothorax OR hemopneumothorax OR hematothorax OR haematothorax OR haemothorax OR hemathorax OR haemathorax OR hemothorax OR ((flail) NEAR/2 (chest* OR thorax OR thoracic* OR sternum*)) OR ((rib* OR costa* OR sternum OR sternal) NEAR/2 (fractur* OR damage* OR injur* OR trauma*)) OR ((heart OR cardiac* OR cardial* OR pericardi* OR percardi*) NEAR/2 (tamponad*)) OR bleeding OR haemorrhage* OR hemorrhage* OR ((blood) NEAR/2 (loss)) OR pneumomediastinum* OR ((mediastin*) NEAR/2 (emphysem*)) OR commotio-cordis)) NOT ((animal* OR rat OR rats OR mouse OR mice OR murine OR dog OR dogs OR canine OR cat OR cats OR feline OR rabbit OR cow OR cows OR bovine OR rodent* OR sheep OR ovine OR pig OR swine OR porcine OR veterinar* OR chick* OR zebrafish* OR baboon* OR nonhuman* OR primate* OR cattle* OR goose OR geese OR duck OR macaque* OR avian* OR bird* OR fish*) NOT (human* OR patient* OR women OR woman OR men OR man))) AND DT=(Article OR Review OR Letter OR Early Access)

Google Scholar (200 records, 108 after removal of duplicates)

resuscitation|CPR|'chest|manual compression'|'heart|cardiac massage' arrest 'thorax|thoracic|heart|cardiac|cardial|lung|pulmonary|chest|vascular injury|damage|contusion|bruise|concussion'|'rib|costa|sternum|sternal fracture|damage|injury'

Conversion of search strategy for use in PubMed

(Resuscitation[mh:NoExp] OR Cardiopulmonary Resuscitation[mh] OR Heart Massage[mh:NoExp] OR resuscit*[tiab] OR reanimat*[tiab] OR CPR[tiab] OR chest-compression*[tiab] OR basic-life-support*[tiab] OR advanced-cardiac-life-support*[tiab] OR BLS[tiab] OR active-compression*[tiab] OR automated-compress*[tiab] OR mechanical-chest-compress*[tiab] OR LUCAS[tiab] OR Autopulse[tiab] OR heart-massag*[tiab] OR cardiac-massag*[tiab] OR manual-compress*[tiab]) AND (Heart Arrest[mh] OR Ventricular Fibrillation[mh:NoExp] OR Arrhythmias, Cardiac[mh:NoExp] OR heart-arrest*[tiab] OR cardiac-arrest*[tiab] OR circulatory-arrest*[tiab] OR standstill*[tiab] OR stand-still*[tiab] OR asystole*[tiab] OR asystolia*[tiab] OR OHCA[tiab] OR IHCA[tiab] OR ((ventrical*[tiab] OR ventricular*[tiab]) AND fibrillat*[tiab]) OR arrhythm*[tiab]) AND (Thoracic Injuries[mh] OR Pneumothorax[mh:NoExp] OR Hemothorax[mh] OR Rib Fractures[mh:NoExp] OR Cardiac Tamponade[mh:NoExp] OR Hemorrhage[mh:NoExp] OR Mediastinal Emphysema[mh:NoExp] OR Vascular System Injuries[mh:NoExp] OR "Resuscitation/adverse effects"[Mesh:NoExp] OR "Heart Massage/adverse effects"[Mesh] OR ((thorax*[tiab] OR thoracic*[tiab] OR heart*[tiab] OR cardiac*[tiab] OR cardial*[tiab] OR lung*[tiab] OR pulmonar*[tiab] OR chest*[tiab] OR cor[tiab] OR myocard*[tiab] OR blood-vessel*[tiab] OR vein*[tiab] OR aorta*[tiab] OR aortic*[tiab] OR artery*[tiab] OR arteries*[tiab] OR arterial*[tiab] OR vascular*[tiab]) AND (injur*[tiab] OR trauma*[tiab] OR damage*[tiab])) OR ((pulmonar*[tiab] OR lung*[tiab] OR cor[tiab] OR myocard*[tiab] OR thorax*[tiab] OR thoracic*[tiab] OR heart*[tiab] OR cardiac*[tiab]) AND (contusion*[tiab] OR bruis*[tiab] OR concussion*[tiab])) OR pneumothorax*[tiab] OR hematopneumothorax*[tiab] OR haematopneumothorax*[tiab] OR haemopneumothorax*[tiab] OR hemopneumothorax*[tiab] OR hematothorax*[tiab] OR haematothorax*[tiab] OR haemothorax*[tiab] OR hemathorax*[tiab] OR haemathorax*[tiab] OR hemothorax*[tiab] OR ((flail*[tiab]) AND (chest*[tiab] OR thorax*[tiab] OR thoracic*[tiab] OR sternum*[tiab])) OR ((rib[tiab] OR costa*[tiab] OR sternum*[tiab] OR sternal*[tiab]) AND (fractur*[tiab] OR damage*[tiab] OR injur*[tiab] OR trauma*[tiab])) OR ((heart*[tiab] OR cardiac*[tiab] OR cardial*[tiab] OR pericardi*[tiab] OR percardi*[tiab]) AND (tamponad*[tiab])) OR bleeding*[tiab] OR haemorrhage*[tiab] OR hemorrhage*[tiab] OR blood-loss*[tiab] OR pneumomediastinum*[tiab] OR ((mediastin*[tiab]) AND (emphysem*[tiab])) OR commotio-cordis*[tiab]) NOT (animals[mh] NOT humans[mh])

Supplementary Online Materials 3: Quality assessment following the MINORS criteria of studies included in systematic review of the prevalence of CPR-related injuries following non-traumatic cardiac arrest

	Clearly stated aim	Inclusion consecutive patients	Prospective data-collection	Appropriate endpoints	Unbiased assessment	Appropriate follow-up	Loss to follow-up <5%	Prospective calculation study size	Adequate control group	Contemporary groups	Baseline equivalence	Adequate statistical analyses	Total score
Adel *et al.* (2022)[1]	1	2	2	1	1	2	2	0	0	2	2	1	16
Azeli *et al.* (2022)[2]	2	2	2	2	1	2	2	0	2	2	2	1	20
Karatasakis *et al.* (2022)[3]	2	2	2	2	2	2	2	0	2	2	1	2	21
Katasako *et al.* (2022)[4]	1	2	0	2	2	2	2	0	2	2	0	2	17
Kawai *et al.* (2022)[5]	2	2	0	2	2	2	2	0	2	2	1	2	19
Kunz *et al.* (2022)[6]	1	2	0	0	1	2	2	0	1	2	1	2	14
Canakci *et al.* (2021)[7]	1	2	0	1	1	2	2	0	2	1	1	1	14
Gaisendrees *et al.* (2021)[8]	2	2	0	2	1	2	2	0	2	2	0	1	16
Hokenek and Erdogan (2021)[9]	2	2	0	2	1	2	2	0	2	2	1	1	17
Karasek *et al.* (2021)[10]	2	2	0	2	1	2	2	0	2	2	1	1	17
Prins *et al.* (2021)[11]	2	2	0	2	2	2	2	0	2	2	1	2	19
Sonnemans *et al.* (2020)[12]	2	2	0	2	0	2	2	0	2	2	1	2	17
Friberg *et al.* (2019)[13]	2	2	0	2	0	2	2	0	2	2	1	1	16
Milling *et al.* (2019)[14]	2	2	0	2	1	2	2	0	2	2	1	2	18
Ondruschka *et al.* (2019)[15]	2	2	0	2	0	2	2	0	2	2	2	1	17
Ondruschka *et al.* (2018)[16]	2	2	0	1	0	2	2	0	2	2	1	2	16

Yusufoglu *et al.* (2018)[17]	2	2	0	2	0	2	2	0	2	0	1	0	13
Beom *et al.* (2017)[18]	1	2	0	2	2	2	2	0	2	0	1	1	15
Koster *et al.* (2017)[19]	2	2	2	2	2	2	2	0	2	2	2	2	22
Oya *et al.* (2016)[20]	2	1	0	1	0	2	2	0	2	0	1	1	12
Seung *et al.* (2016)[21]	2	1	0	2	1	2	2	0	2	2	2	2	18
Vahedian-Azimi *et al.* (2016)[22]	1	1	2	2	2	2	1	0	2	2	2	1	18
Koga *et al.* (2015)[23]	2	1	0	2	0	2	2	0	2	2	2	1	16
Lardi *et al.* (2015)[24]	2	2	0	2	0	2	2	0	2	2	1	1	16
Štěchovský *et al.* (2015)[25]	1	1	1	2	0	2	2	0	2	2	0	0	13
Smekal *et al.* (2014)[26]	2	2	2	1	1	2	2	0	2	2	2	2	20
Pinto *et al.* (2013)[27]	1	1	0	1	0	2	2	0	2	2	0	1	12
Charaschaisri *et al.* (2011)[28]	1	2	0	2	1	2	2	0	2	2	2	1	17
Smekal *et al.* (2009)[29]	2	1	2	2	2	2	2	0	2	2	2	1	20
Oschatz *et al.* (2001)[30]	2	2	1	1	2	2	2	0	1	2	1	1	17
Baubin *et al.* (1999)[31]	1	1	2	2	0	2	1	1	2	2	2	1	17
Rabl *et al.* (1996)[32]	0	1	0	2	0	2	1	0	2	2	1	0	11
Cohen *et al.* (1993)[33]	2	2	2	2	2	2	2	2	2	2	2	1	23
Hwang *et al.* (2021)[34]	1	2	0	1	2	2	2	0	N.A.	N.A.	N.A.	N.A.	10
Moriguchi *et al.* (2021)[35]	2	2	0	2	0	2	2	0	N.A.	N.A.	N.A.	N.A.	10
Jang *et al.* (2020)[36]	2	2	0	2	0	2	2	0	N.A.	N.A.	N.A.	N.A.	10
Kim *et al.* (2020)[37]	2	2	0	2	2	2	2	0	N.A.	N.A.	N.A.	N.A.	12
Milling *et al.* (2020)[38]	2	2	1	2	0	2	2	0	N.A.	N.A.	N.A.	N.A.	11
Oh and Kim (2020)[39]	2	2	0	2	0	2	2	0	N.A.	N.A.	N.A.	N.A.	10
Viniol *et al.* (2020)[40]	1	2	0	1	0	2	2	0	N.A.	N.A.	N.A.	N.A.	8

Zaidi *et al.* (2020)[41]	2	2	0	2	1	2	2	0	N.A.	N.A.	N.A.	N.A.	11
Zotzmann *et al.* (2020)[42]	1	2	1	1	1	2	2	0	N.A.	N.A.	N.A.	N.A.	10
Azeli *et al.* (2019)[43]	2	2	0	2	0	2	2	0	N.A.	N.A.	N.A.	N.A.	10
Deliliga *et al.* (2019)[44]	1	2	0	1	1	2	2	0	N.A.	N.A.	N.A.	N.A.	9
Iglesies *et al.* (2019)[45]	1	2	2	1	1	2	2	0	N.A.	N.A.	N.A.	N.A.	11
Dunham *et al.* (2018)[46]	2	1	0	2	1	2	2	0	N.A.	N.A.	N.A.	N.A.	10
Setälä *et al.* (2018)[47]	2	2	2	2	1	2	2	0	N.A.	N.A.	N.A.	N.A.	13
Takayama *et al.* (2018)[48]	2	2	0	1	0	2	2	0	N.A.	N.A.	N.A.	N.A.	9
Cha *et al.* (2017)[49]	1	2	0	2	2	2	2	0	N.A.	N.A.	N.A.	N.A.	11
Nomura *et al.* (2017)[50]	0	2	0	1	0	1	2	0	N.A.	N.A.	N.A.	N.A.	6
Yamaguchi *et al.* (2017)[51]	2	1	0	1	1	2	2	0	N.A.	N.A.	N.A.	N.A.	9
Ihnát Rudinská *et al.* (2016)[52]	2	2	1	1	0	2	2	0	N.A.	N.A.	N.A.	N.A.	10
Boland *et al.* (2015)[53]	1	1	0	2	1	2	2	0	N.A.	N.A.	N.A.	N.A.	9
Kaldırım *et al.* (2015)[54]	1	1	0	2	0	2	2	0	N.A.	N.A.	N.A.	N.A.	8
Kashiwagi *et al.* (2015)[55]	2	1	0	1	1	2	2	0	N.A.	N.A.	N.A.	N.A.	9
Kralj *et al.* (2015)[56]	2	2	0	2	0	2	2	0	N.A.	N.A.	N.A.	N.A.	10
Choi *et al.* (2014)[57]	2	2	0	2	1	2	2	0	N.A.	N.A.	N.A.	N.A.	11
Cho *et al.* (2013)[58]	2	1	0	2	2	2	1	0	N.A.	N.A.	N.A.	N.A.	10
Hellevuo *et al.* (2013)[59]	2	1	2	2	2	2	2	0	N.A.	N.A.	N.A.	N.A.	13
Kim *et al.* (2013)[60]	1	1	1	1	0	2	0	0	N.A.	N.A.	N.A.	N.A.	6
Smekal *et al.* (2013)[61]	2	1	1	2	1	2	2	0	N.A.	N.A.	N.A.	N.A.	11
Kim *et al.* (2011)[62]	1	1	0	1	2	2	2	0	N.A.	N.A.	N.A.	N.A.	9
Meron *et al.* (2007)[63]	1	0	0	2	0	1	2	0	N.A.	N.A.	N.A.	N.A.	6
Nishida *et al.* (2006)[64]	1	0	1	2	0	2	2	0	N.A.	N.A.	N.A.	N.A.	8

Black *et al.* (2004)[65]	2	1	0	2	0	2	2	0	N.A.	N.A.	N.A.	N.A.	9
Lederer *et al.* (2004)[66]	2	1	1	2	0	2	2	0	N.A.	N.A.	N.A.	N.A.	10
Bedell and Fulton(1986)[67]	2	2	0	1	0	2	2	0	N.A.	N.A.	N.A.	N.A.	9
Powner *et al.* (1984)[68]	2	1	0	1	1	2	2	0	N.A.	N.A.	N.A.	N.A.	9
Bjork *et al.* (1982)[69]	1	2	1	2	0	2	2	0	N.A.	N.A.	N.A.	N.A.	10
Murtomaa and Korttila (1974)[70]	2	2	2	1	0	2	2	0	N.A.	N.A.	N.A.	N.A.	11
Anthony and Tattersfield (1969)[71]	1	2	0	1	0	2	2	0	N.A.	N.A.	N.A.	N.A.	8
Saphir (1968)[72]	1	2	2	1	1	2	2	0	N.A.	N.A.	N.A.	N.A.	11
Lundberg *et al.* (1967)[73]	1	2	0	1	0	2	2	0	N.A.	N.A.	N.A.	N.A.	8
Minuck (1966)[74]	1	2	0	1	1	2	2	0	N.A.	N.A.	N.A.	N.A.	9
SSRF studies													
Prins *et al.* (2022)[75]	2	2	0	2	1	2	2	0	2	2	1	2	18
DeVoe *et al.* (2022)[76]	1	2	0	2	2	2	2	0	N.A.	N.A.	N.A.	N.A.	11
Claydon *et al.* (2020)[77]	1	1	0	1	1	2	2	0	N.A.	N.A.	N.A.	N.A.	8
Lee *et al.* (2020)[78]	1	0	0	1	2	2	2	0	N.A.	N.A.	N.A.	N.A.	8
Drahos *et al.* (2019)[79]	1	0	0	2	2	2	2	0	N.A.	N.A.	N.A.	N.A.	9
Pouwels *et al.* (2018)[80]	1	0	0	2	2	2	2	0	N.A.	N.A.	N.A.	N.A.	9
Ananiadou *et al.* (2010)[81]	1	0	0	1	1	2	2	0	N.A.	N.A.	N.A.	N.A.	7

CPR, cardiopulmonary resuscitation; N.A., not applicable for studies without a control group

Supplementary Online Materials 4: Forest plots for any CPR-related injury and thoracic injuries

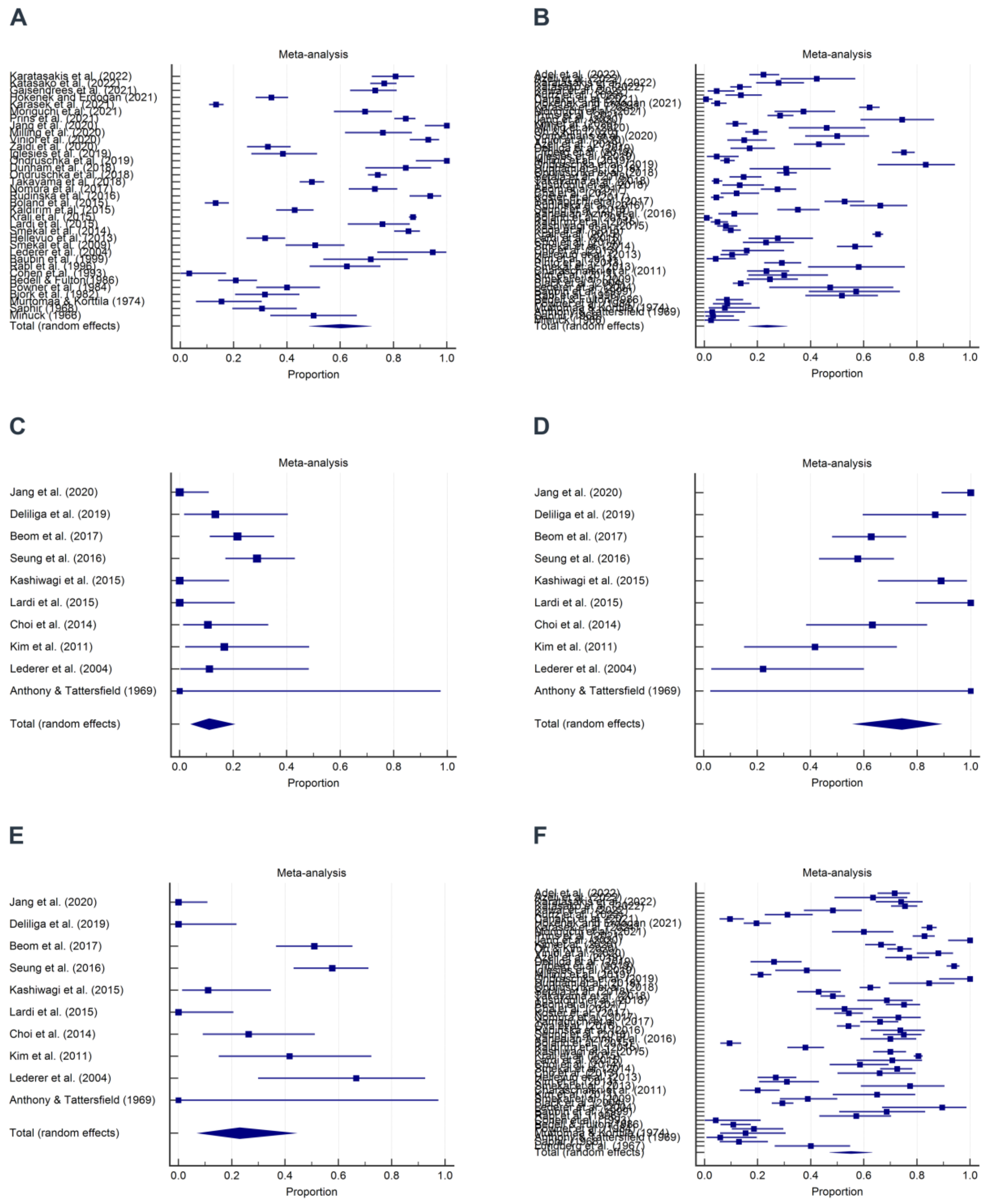

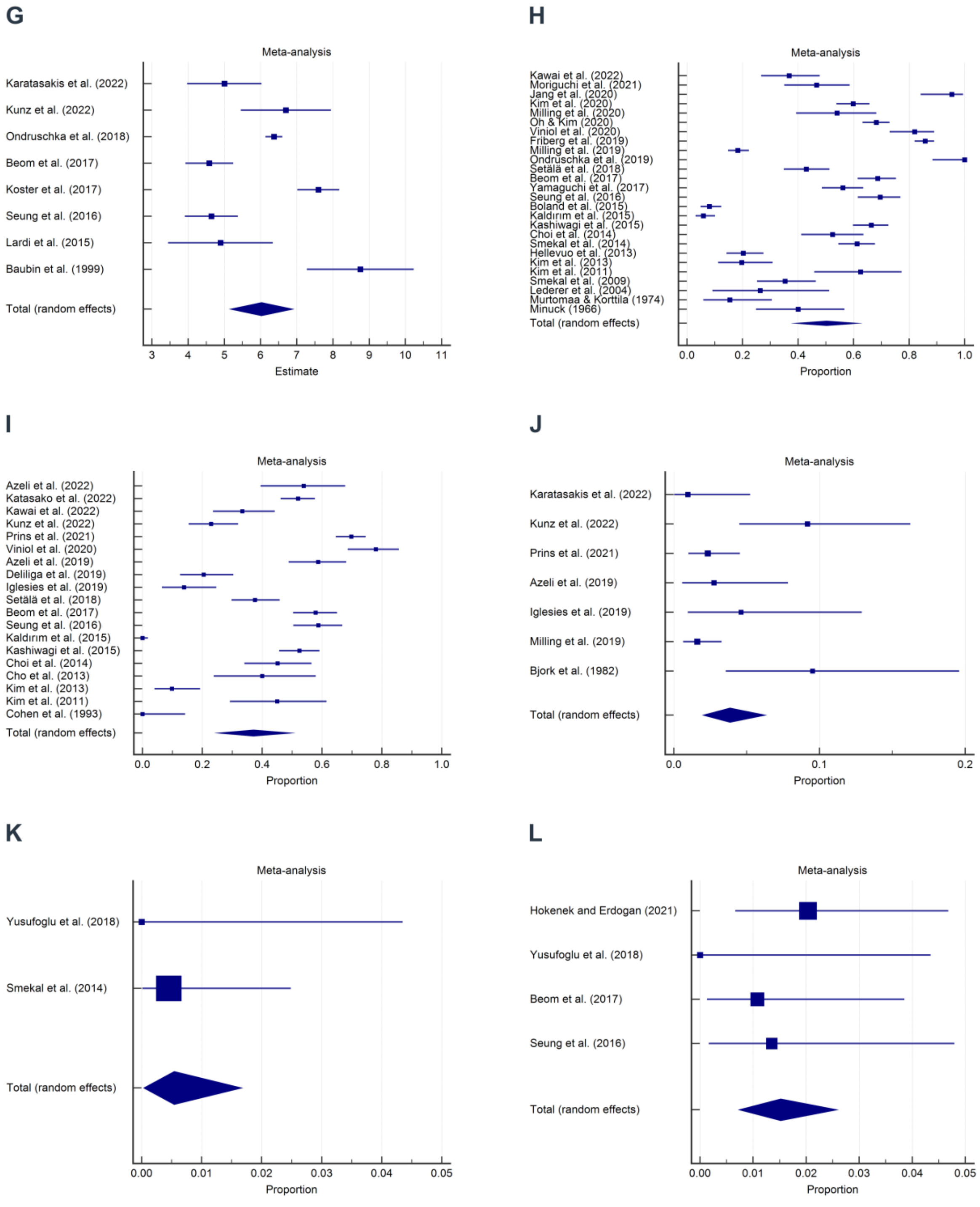
G
Meta-analysis
Karatasakis et al. (2022)
Kunz et al. (2022)
Ondruschka et al. (2018)
Beom et al. (2017)
Koster et al. (2017)
Seung et al. (2016)
Lardi et al. (2015)
Baubin et al. (1999)
Total (random effects)
3 4 5 6 7 8 9 10 11
Estimate
H
Meta-analysis
Kawai et al. (2022)
Moriguchi et al. (2021)
Jang et al. (2020)
Kim et al. (2020)
Milling et al. (2020)
Oh & Kim (2020)
Viniol et al. (2020)
Friberg et al. (2019)
Milling et al. (2019)
Ondruschka et al. (2019)
Setälä et al. (2018)
Beom et al. (2017)
Yamaguchi et al. (2017)
Seung et al. (2016)
Boland et al. (2015)
Kaldırım et al. (2015)
Kashiwagi et al. (2015)
Choi et al. (2014)
Smekal et al. (2014)
Hellevuo et al. (2013)
Kim et al. (2013)
Kim et al. (2011)
Smekal et al. (2009)
Lederer et al. (2004)
Murtomaa & Korttila (1974)
Minuck (1966)
Total (random effects)
0.0 0.2 0.4 0.6 0.8 1.0
Proportion
I
Meta-analysis
Azeli et al. (2022)
Katasako et al. (2022)
Kawai et al. (2022)
Kunz et al. (2022)
Prins et al. (2021)
Viniol et al. (2020)
Azeli et al. (2019)
Deliliga et al. (2019)
Iglesies et al. (2019)
Setälä et al. (2018)
Beom et al. (2017)
Seung et al. (2016)
Kaldırım et al. (2015)
Kashiwagi et al. (2015)
Choi et al. (2014)
Cho et al. (2013)
Kim et al. (2013)
Kim et al. (2011)
Cohen et al. (1993)
Total (random effects)
0.0 0.2 0.4 0.6 0.8 1.0
Proportion
J
Meta-analysis
Karatasakis et al. (2022)
Kunz et al. (2022)
Prins et al. (2021)
Azeli et al. (2019)
Iglesies et al. (2019)
Milling et al. (2019)
Bjork et al. (1982)
Total (random effects)
0.0 0.1 0.2
Proportion
K
Meta-analysis
Yusufoglu et al. (2018)
Smekal et al. (2014)
Total (random effects)
0.00 0.01 0.02 0.03 0.04 0.05
Proportion
L
Meta-analysis
Hokenek and Erdogan (2021)
Yusufoglu et al. (2018)
Beom et al. (2017)
Seung et al. (2016)
Total (random effects)
0.00 0.01 0.02 0.03 0.04 0.05
Proportion

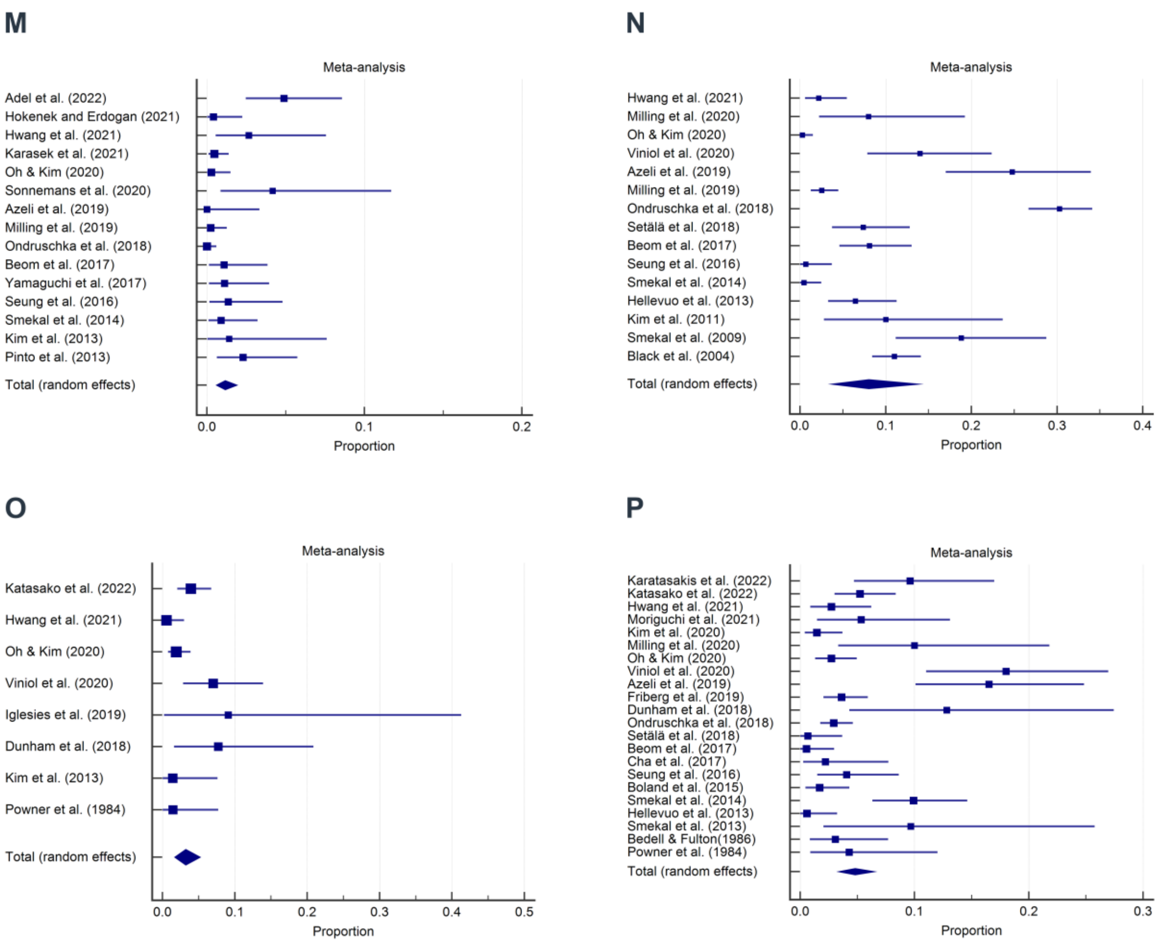

A, any CPR-related injury; B, any sternum fracture C, sternum fracture in the upper third of the sternum; D, sternum fracture in the middle third of the sternum; E, sternum fracture in the lower third of the sternum; F, any rib fracture; G, rib fracture mean; H, multiple rib fractures; I, bilateral rib fractures; J, flail chest; K, clavicle fracture; L, scapula fracture; M, vertebral injury; N, extrathoracic chest wall injury; O, pneumomediastinum; P, hemomediastinum.

CPR, cardiopulmonary resuscitation.

Supplementary Online Materials 5: Forest plots for CPR-related pulmonary injuries

A

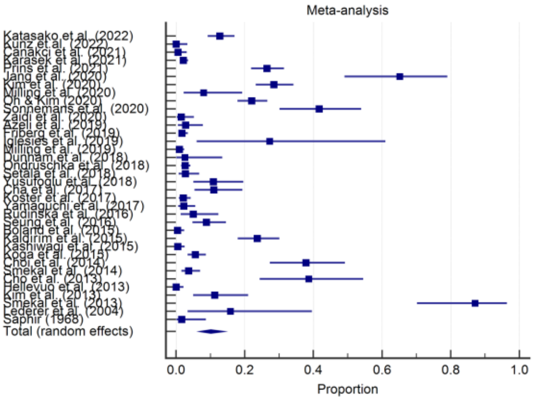

B

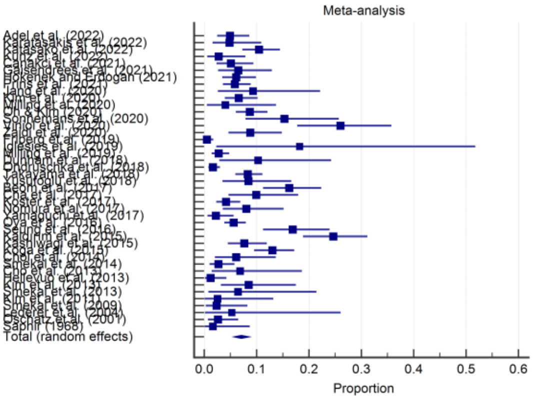

C

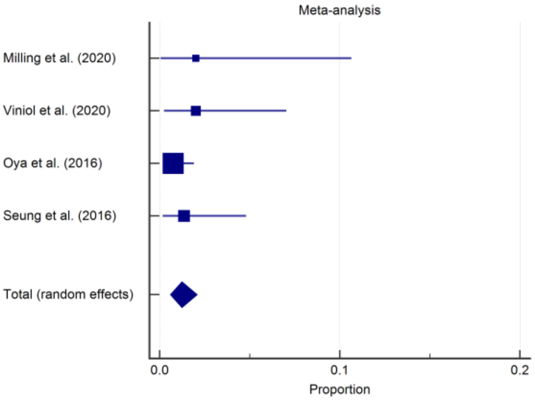

D

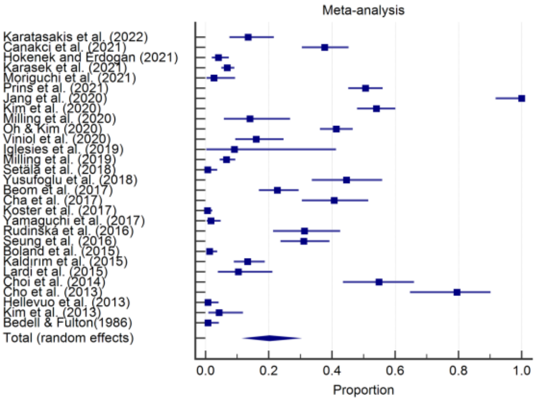

E

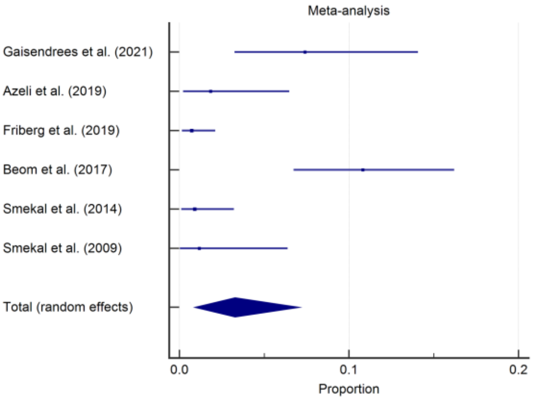

F

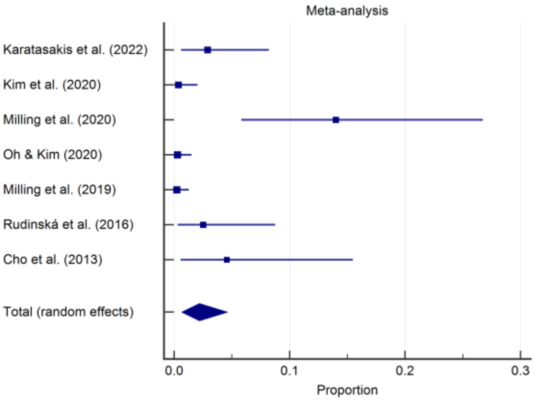

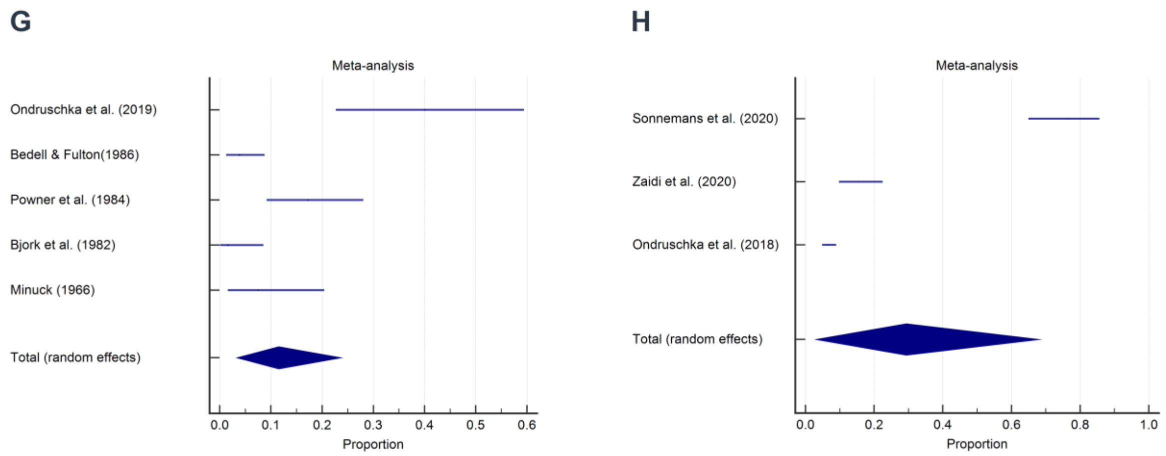

A, hemothorax; B, pneumothorax; C tension pneumothorax; D, pulmonary contusion; E, pulmonary hematoma; F, pulmonary laceration; G, bone marrow or fat embolism; H, other pulmonary injury.

CPR, cardiopulmonary resuscitation

Supplementary Online Materials 6: Forest plots for CPR-related cardiac injuries

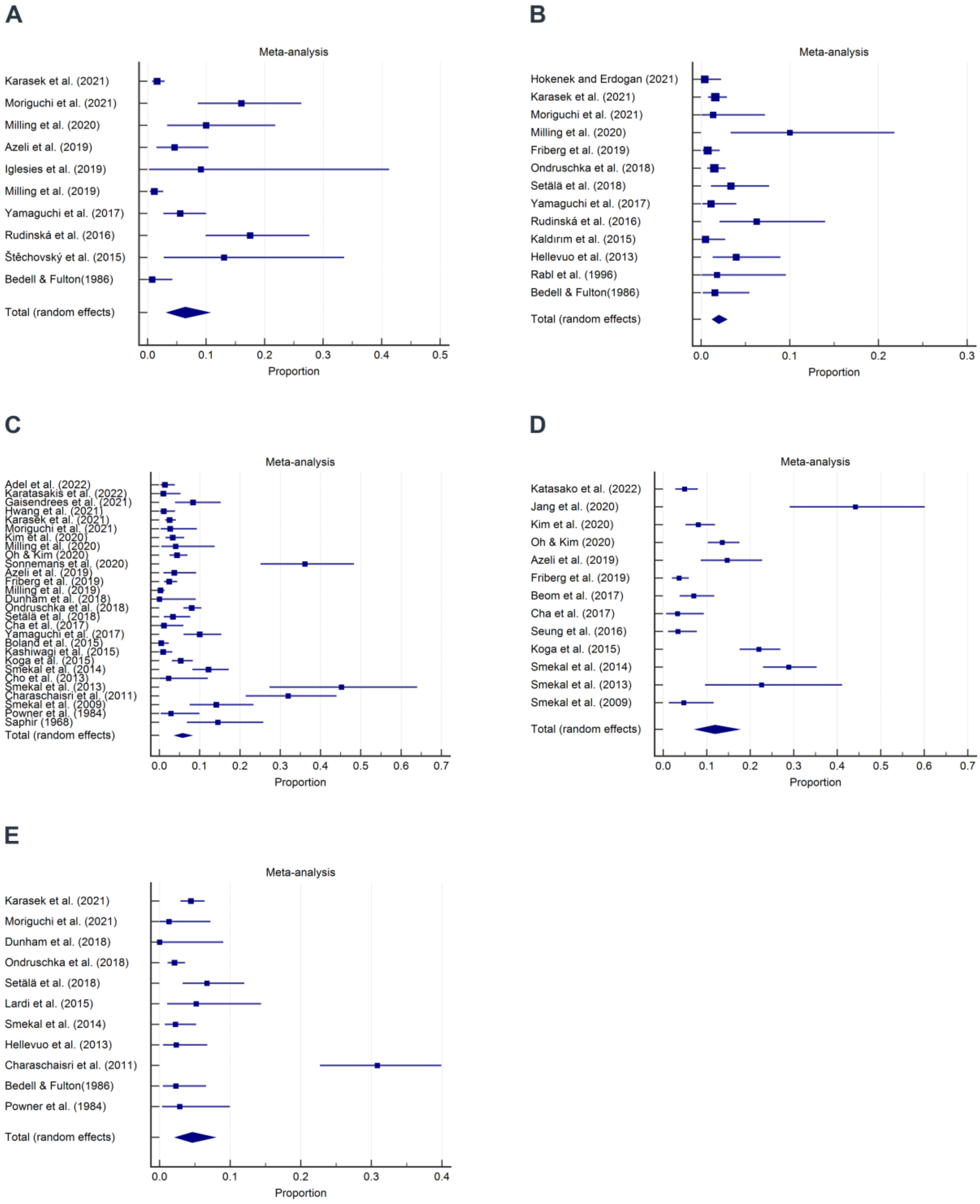

A, cardiac contusion; B, cardiac laceration, rupture or perforation; C, pericardial or epicardial injury; D, retrosternal hematoma; E, other cardiac injury.

CPR, cardiopulmonary resuscitation.

Supplementary Online Materials 7: Forest plots for CPR-related abdominal injuries

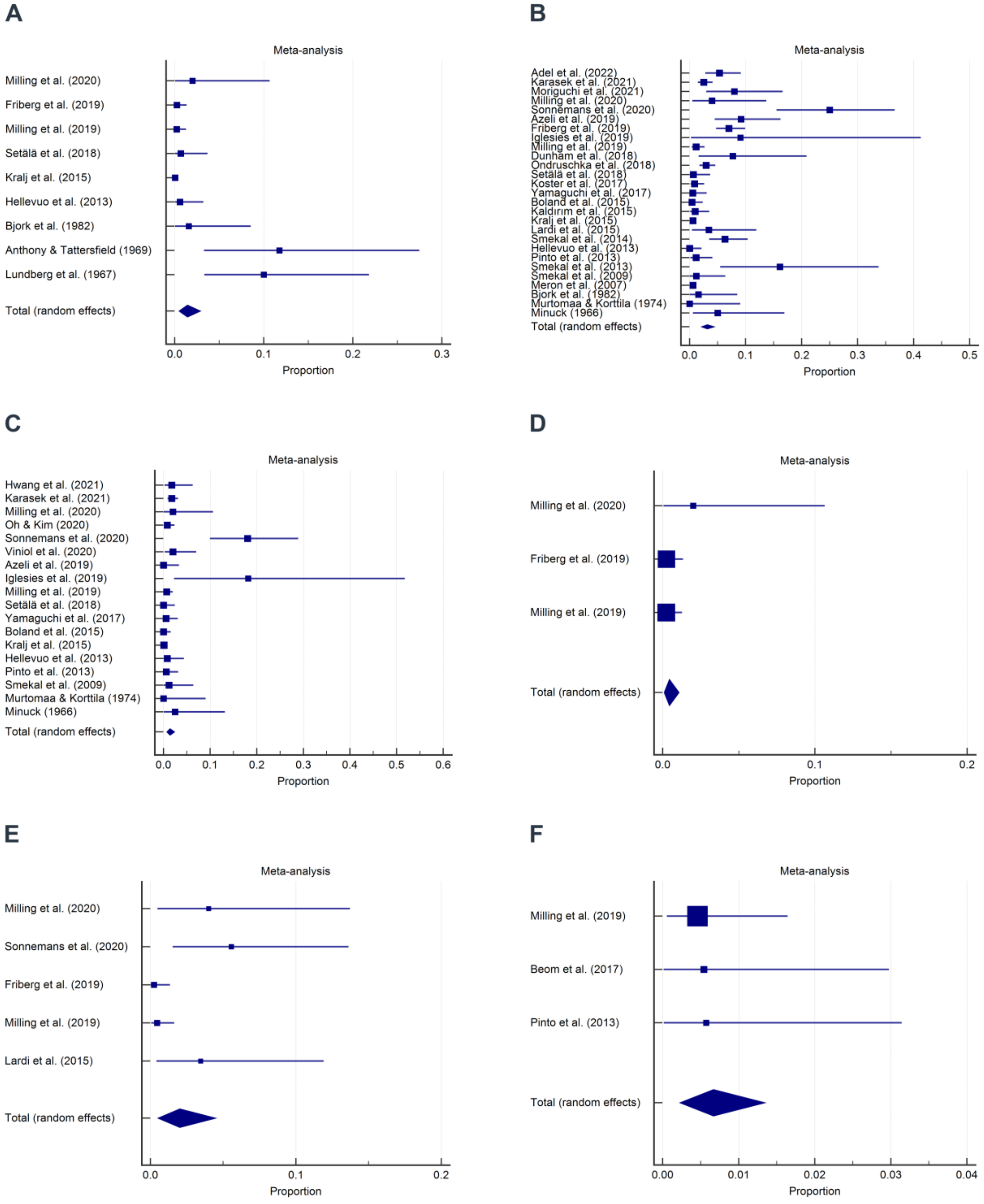

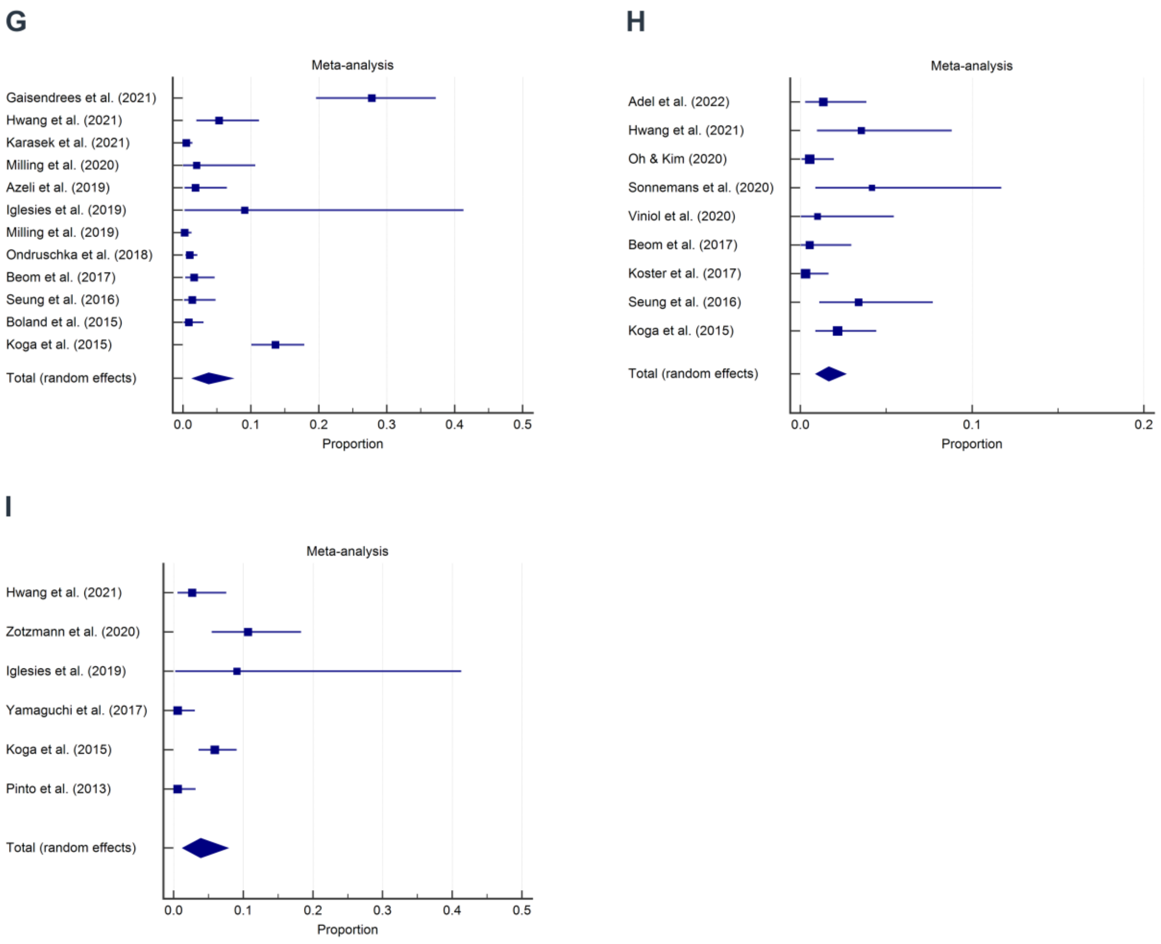

A, stomach injury; B, liver injury; C, spleen injury; D, pancreas injury; E, kidney injury; F, intestinal injury; G, hemoperitoneum; H, pneumoperitoneum; I, other abdominal injury

CPR, cardiopulmonary resuscitation.

Supplementary Online Materials 8: Forest plots for other CPR-related injuries

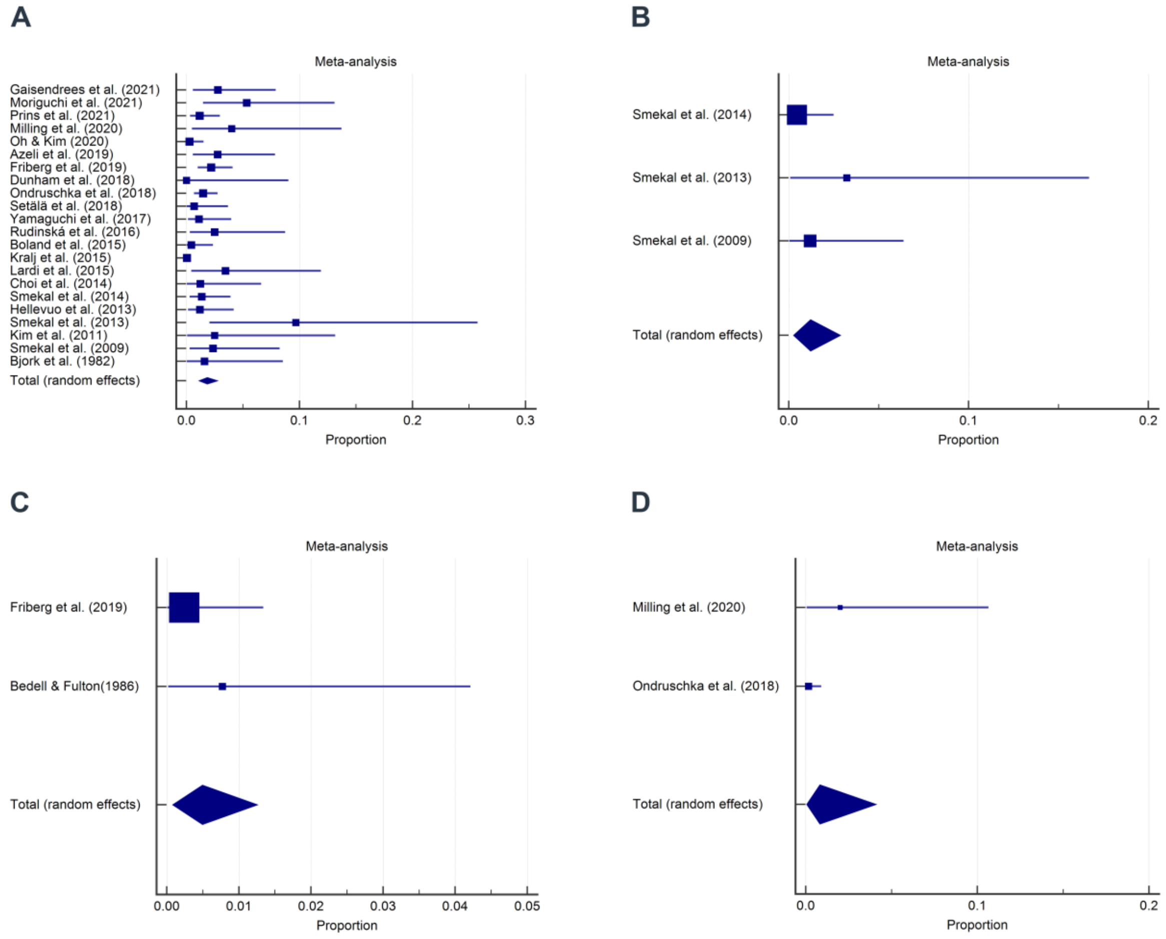

A, thoracic vascular injury; B, abdominal aorta injury; C, trachea injury; D, diaphragm injury.

CPR, cardiopulmonary resuscitation.

Supplementary Online Materials 9: Funnel plots for any CPR-related injury and thoracic injuries

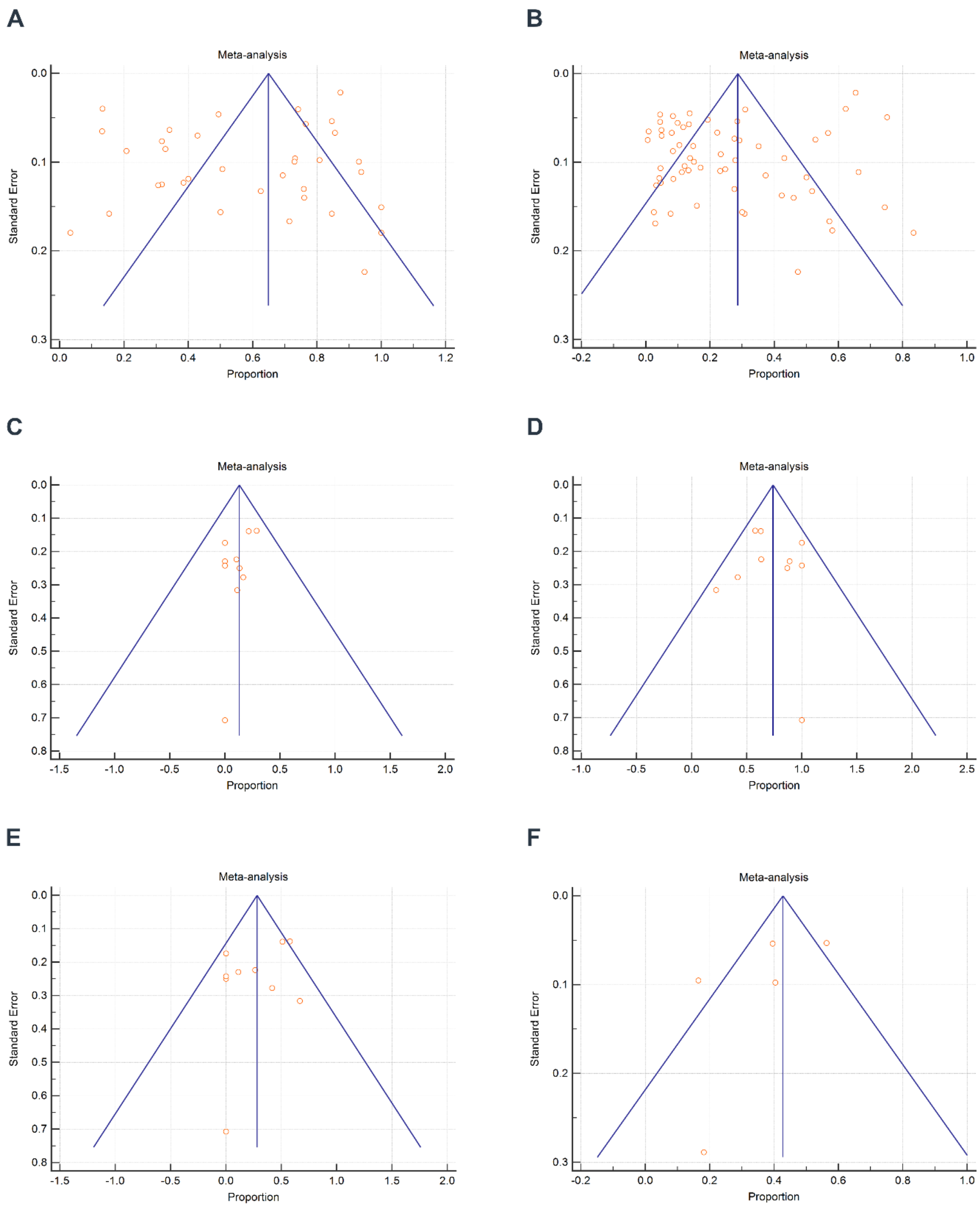

G

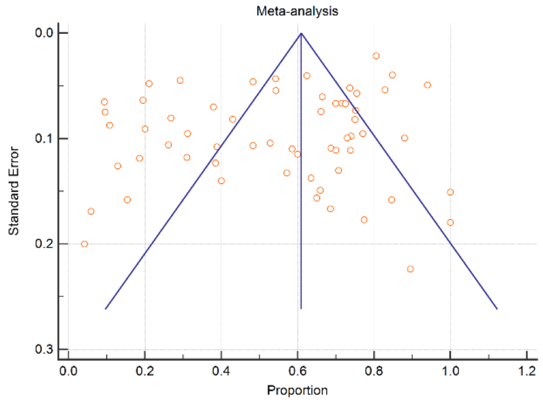
Meta-analysis
Standard Error
0.0
0.1
0.2
0.3
0.0
0.2
0.4
0.6
0.8
1.0
1.2
Proportion

H

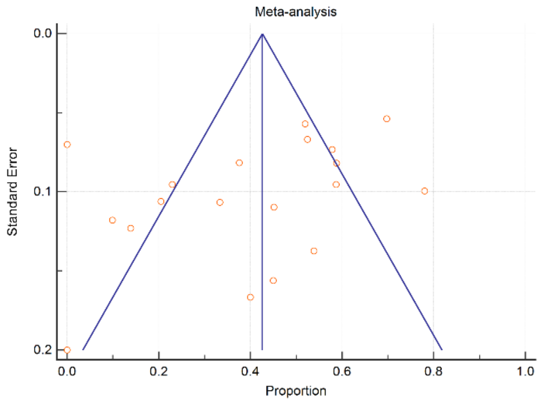
Meta-analysis
Standard Error
0.0
0.1
0.2
0.0
0.2
0.4
0.6
0.8
1.0
Proportion

I

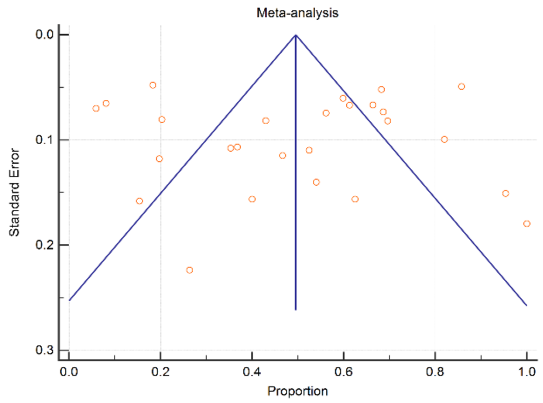
Meta-analysis
Standard Error
0.0
0.1
0.2
0.3
0.0
0.2
0.4
0.6
0.8
1.0
Proportion

J

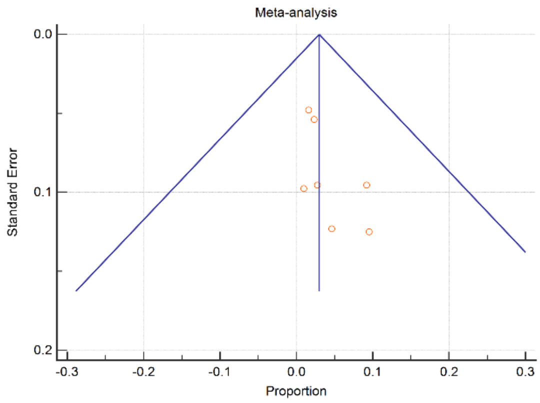
Meta-analysis
Standard Error
0.0
0.1
0.2
-0.3
-0.2
-0.1
0.0
0.1
0.2
0.3
Proportion

K

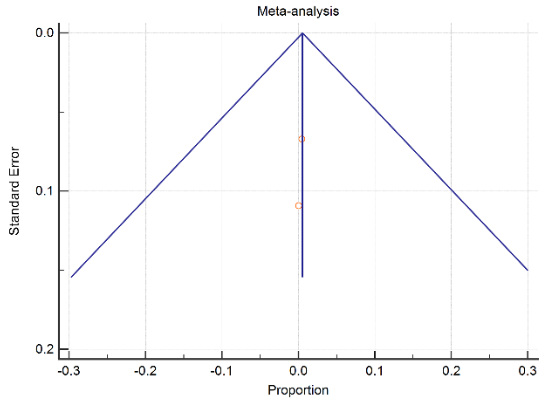
Meta-analysis
Standard Error
0.0
0.1
0.2
-0.3
-0.2
-0.1
0.0
0.1
0.2
0.3
Proportion

L

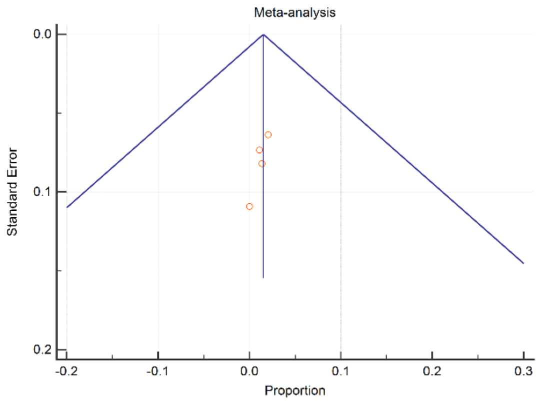
Meta-analysis
Standard Error
0.0
0.1
0.2
-0.2
-0.1
0.0
0.1
0.2
0.3
Proportion

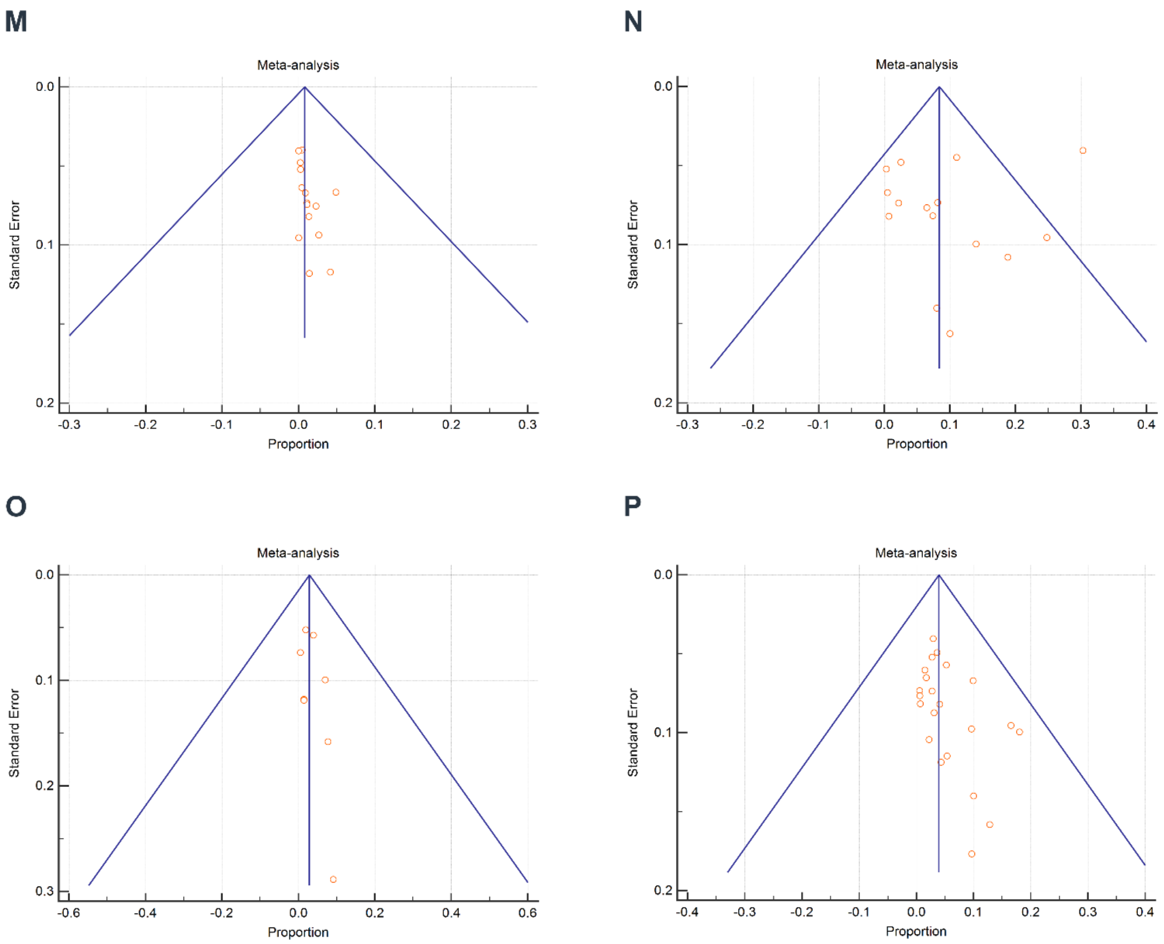

A, any CPR-related injury; B, any sternum fracture C, sternum fracture in the upper third of the sternum; D, sternum fracture in the middle third of the sternum; E, sternum fracture in the lower third of the sternum; F, flail sternum; G, any rib fracture; H, bilateral rib fractures; I, multiple rib fractures; J, flail chest; K, clavicle fracture; L, scapula fracture; M, vertebral injury; N, extrathoracic chest wall injury; O, pneumomediastinum; P, hemomediastinum.

CPR, cardiopulmonary resuscitation.

Supplementary Online Materials 10: Funnel plots for CPR-related pulmonary injuries

A

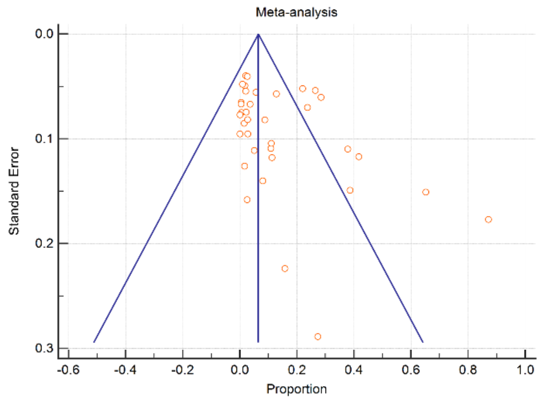

B

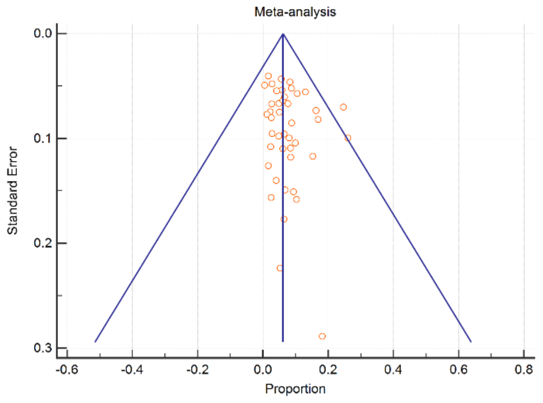

C

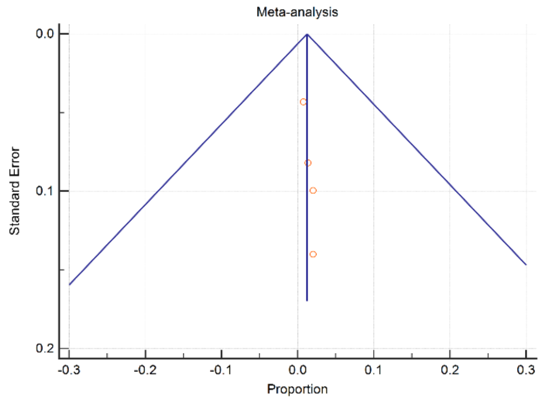

D

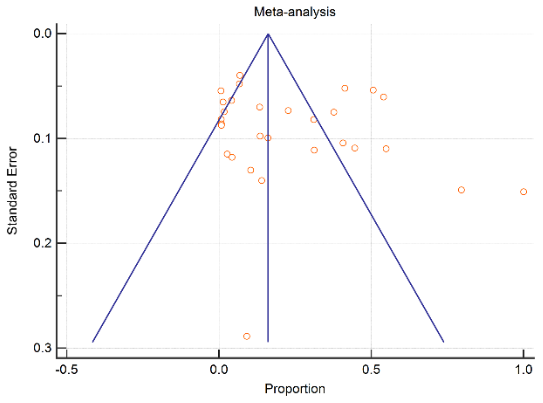

E

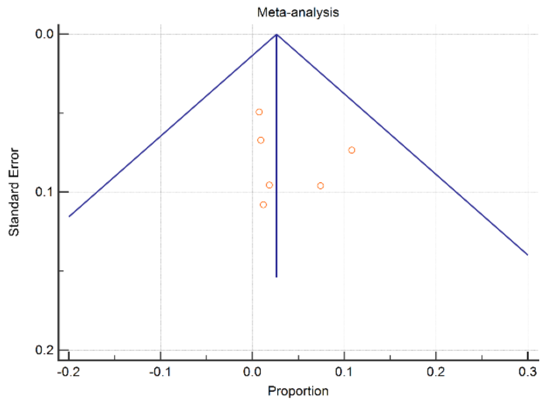

F

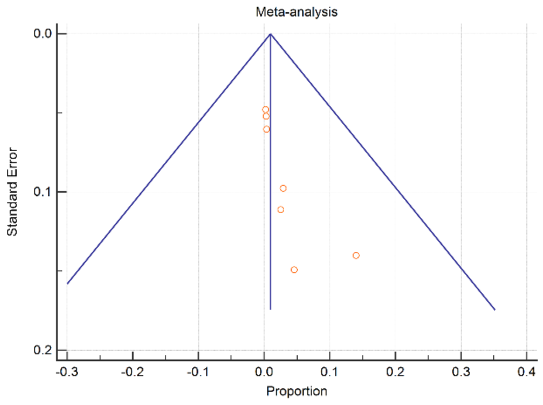

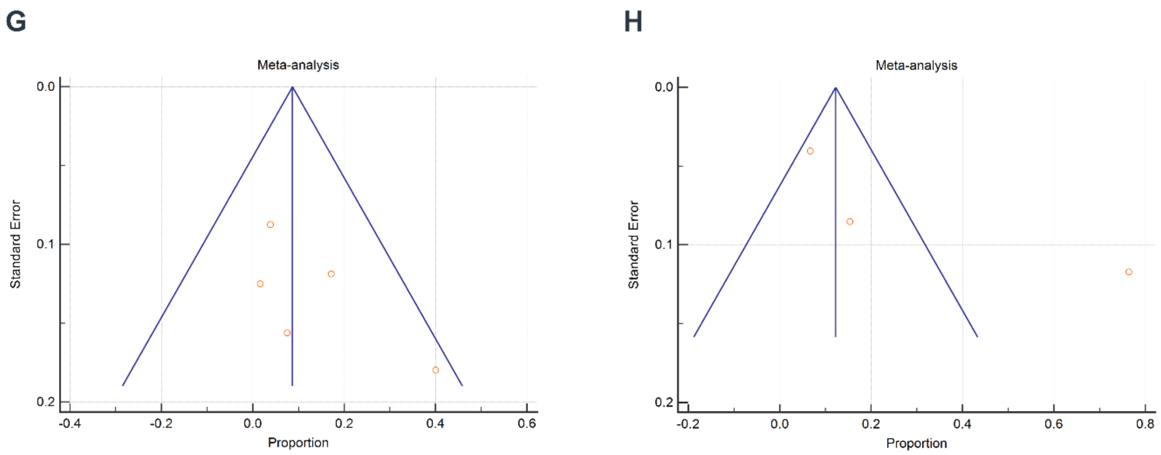

A, hemothorax; B, pneumothorax; C tension pneumothorax; D, pulmonary contusion; E, pulmonary hematoma; F, pulmonary laceration; G, bone marrow or fat embolism; H, other pulmonary injury.

CPR, cardiopulmonary resuscitation.

Supplementary Online Materials 11: Funnel plots for CPR-related cardiac injuries

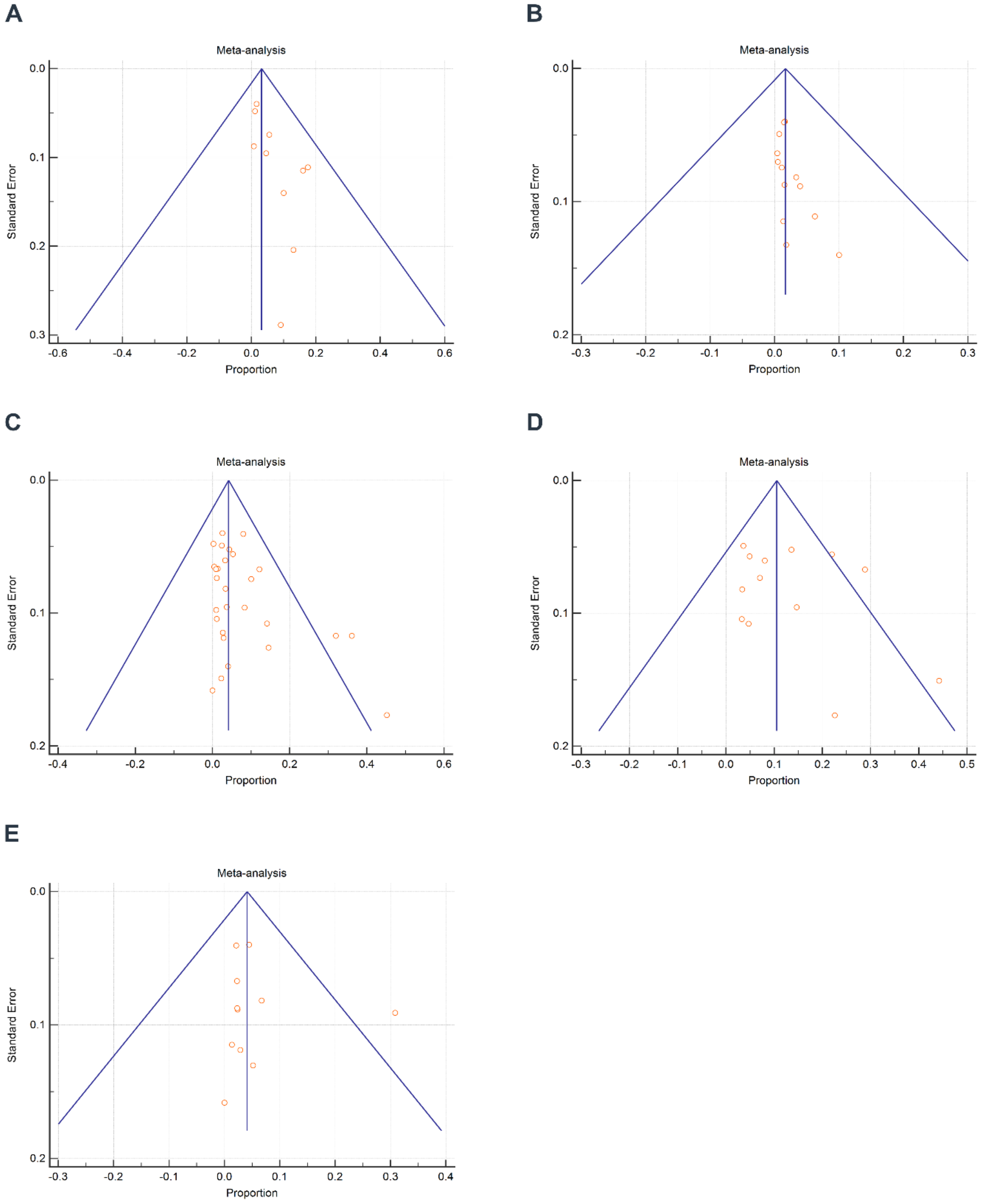

A, cardiac contusion; B, cardiac laceration, rupture or perforation; C, pericardial or epicardial injury; D, retrosternal hematoma; E, other cardiac injury.

CPR, cardiopulmonary resuscitation.

Supplementary Online Materials 12: Funnel plots for CPR-related abdominal injuries

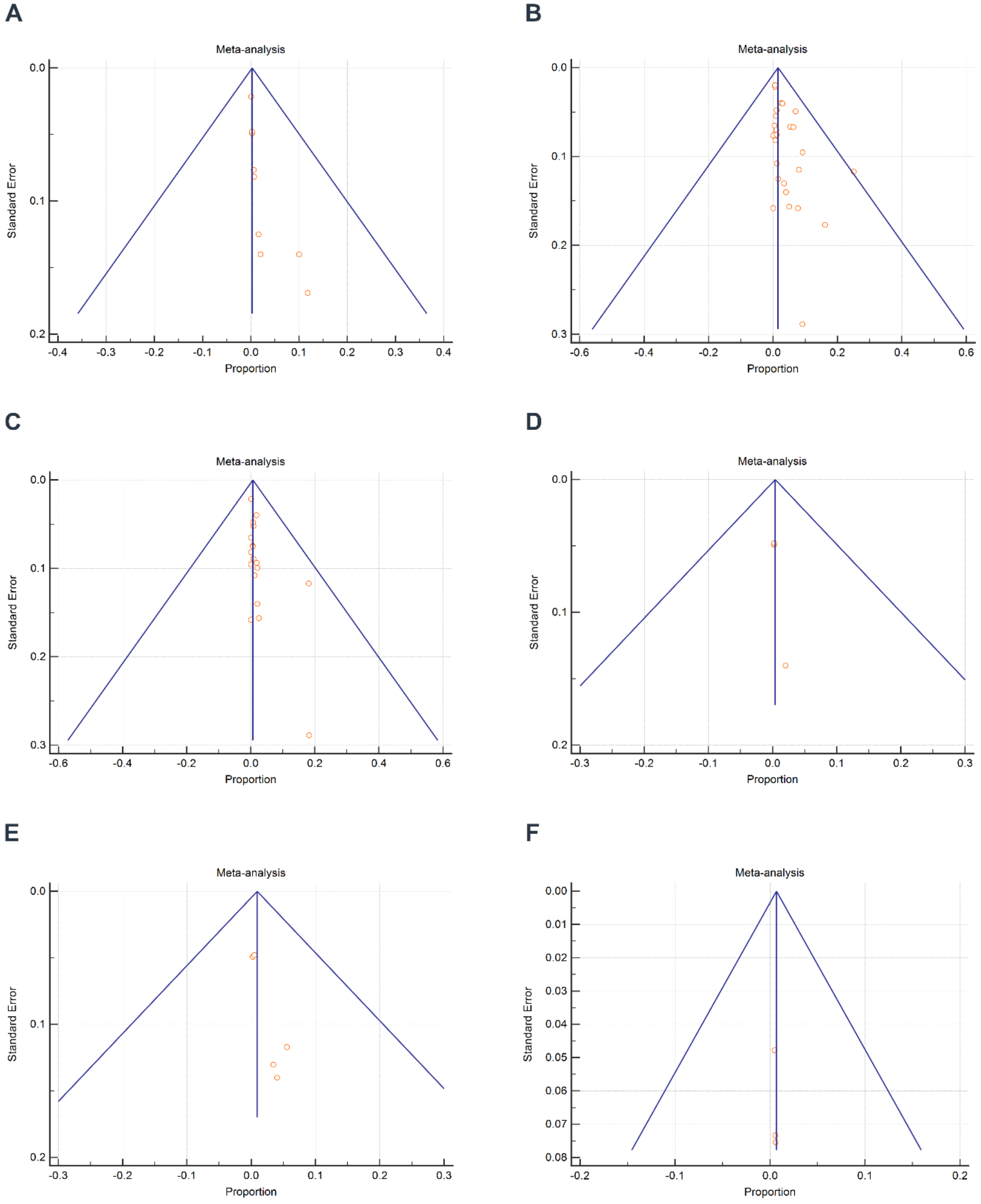

A, stomach injury; B, liver injury; C, spleen injury; D, pancreas injury; E, kidney injury; F, intestinal injury; G, hemoperitoneum; H, pneumoperitoneum; I, other abdominal injury

CPR, cardiopulmonary resuscitation.

Supplementary Online Materials 13: Funnel plots for other CPR-related injuries

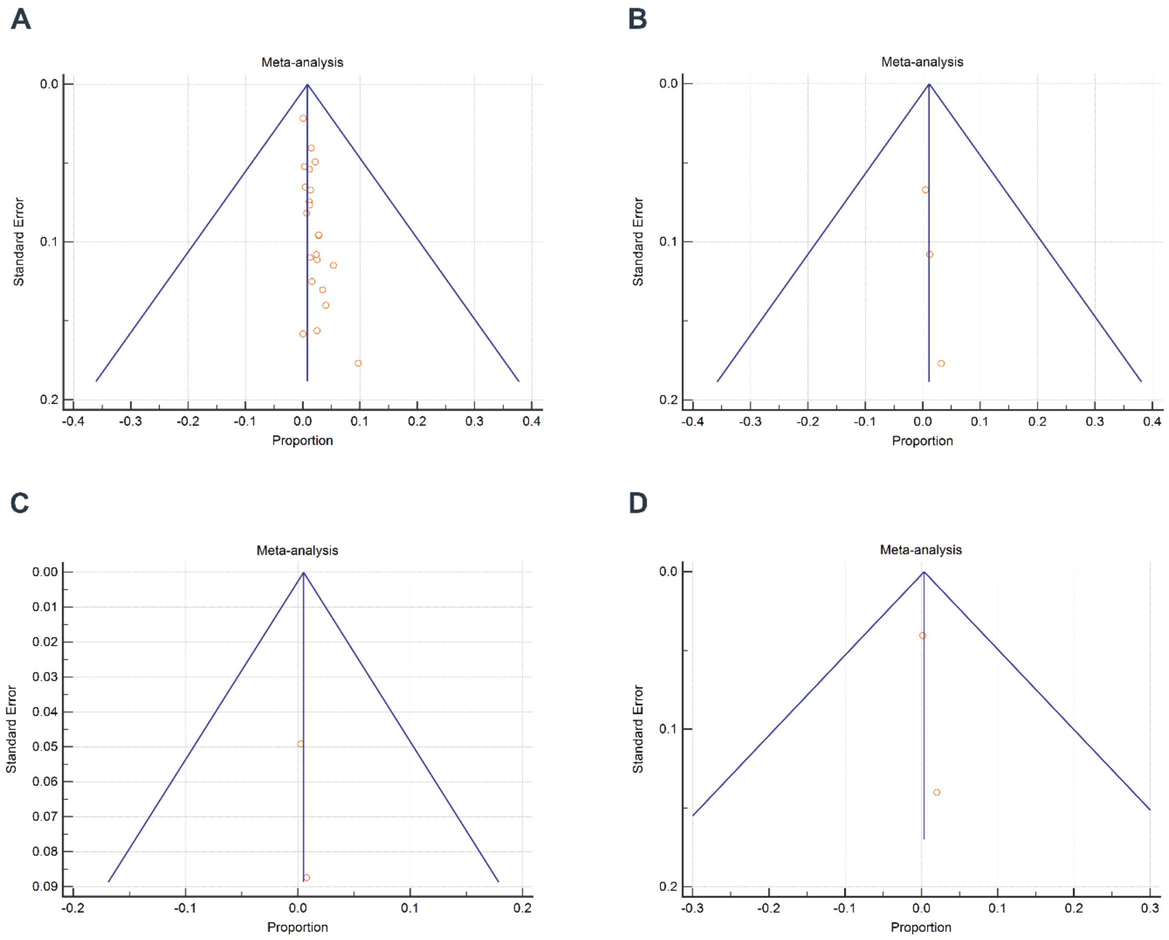

A, thoracic vascular injury; B, abdominal aorta injury; C, trachea injury; D, diaphragm injury.

CPR, cardiopulmonary resuscitation.

Supplementary Online Materials 14: Forest plots detailing the risk ratio for CPR-related thoracic injury of manual versus mechanical CPR following non-traumatic cardiac arrest

A

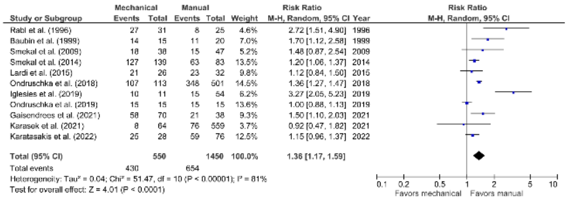

B

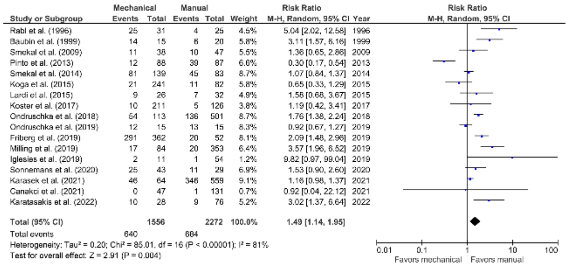

C

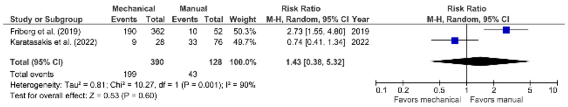

D

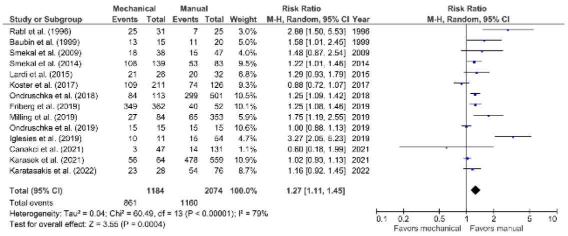

E

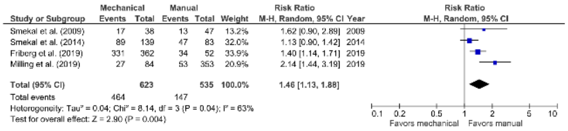

F

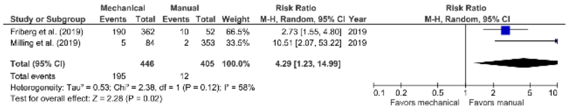

G

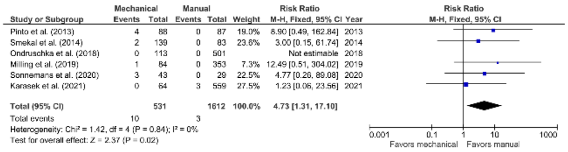

H

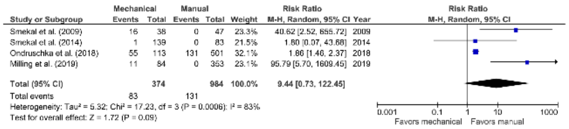

I

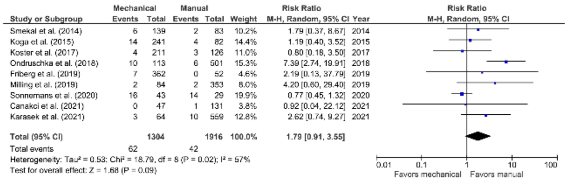

J

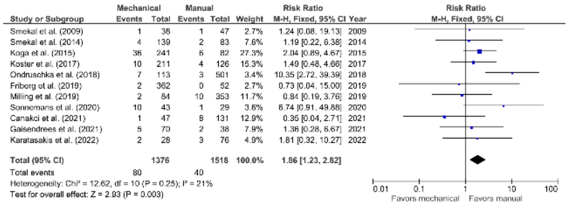

K

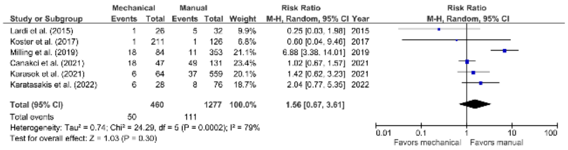

L

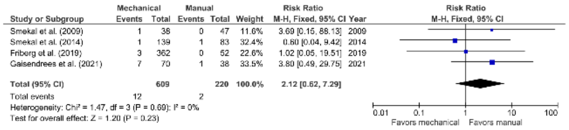

M

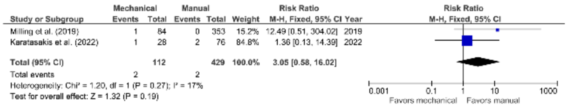

N

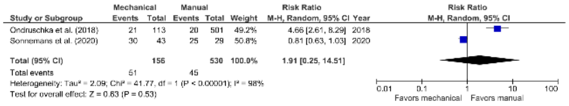

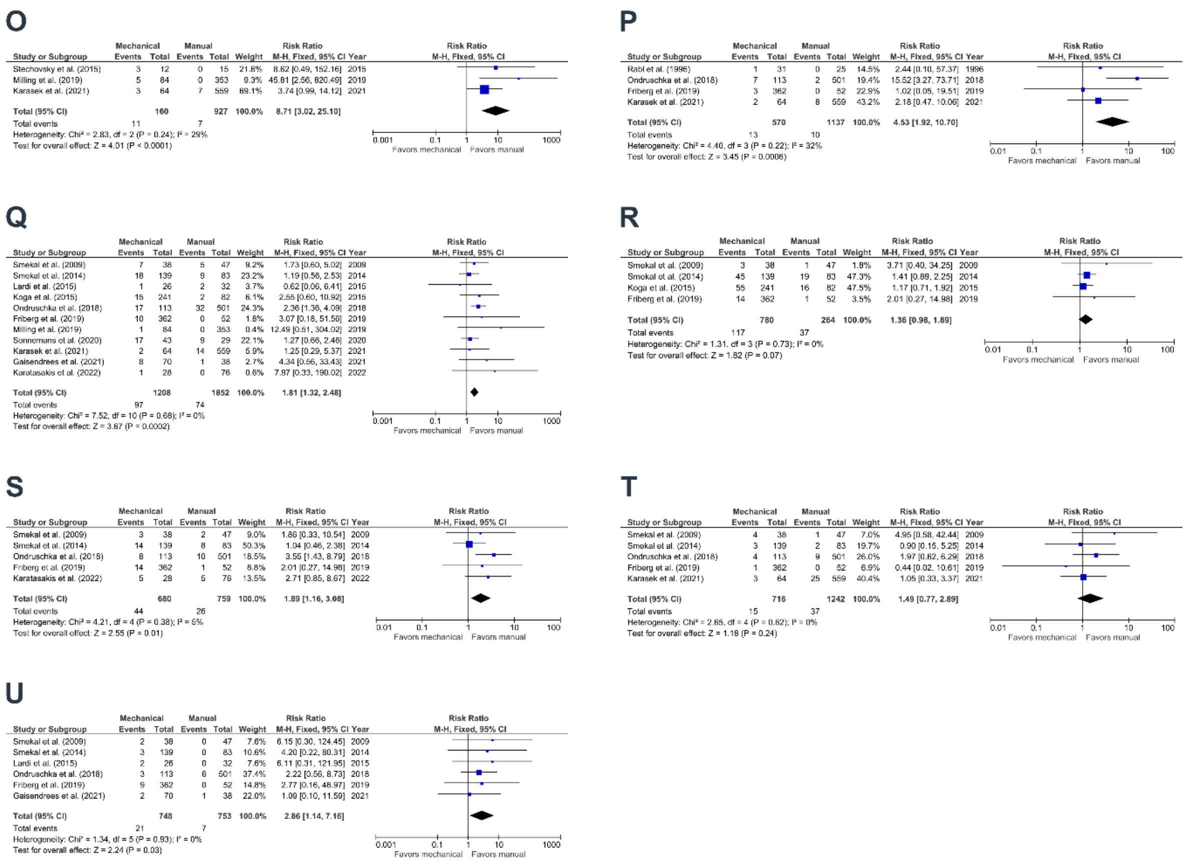

An intervention associated with less risk of CPR-related injury is favored.

A, any CPR-related injury; B, sternum fracture; C, flail sternum; D, rib fracture; E, multiple rib fractures; F, flail chest; G, vertebral fracture; H, extrathoracic chest wall injury; I, hemothorax; J, pneumothorax; K, pulmonary contusion; L, pulmonary hematoma; M, pulmonary laceration; N, other pulmonary injury; O, myocardial contusion; P, cardiac laceration, rupture, or perforation; Q, pericardial or epicardial injury; R, retrosternal hematoma; S, hemomediastinum; T, other cardiac injury; U, thoracic vascular injury.

CI, confidence interval; M-H, Mantel-Haenszel; SD, standard deviation.

Supplementary Online Materials 15: Forest plots detailing the risk ratio for CPR-related abdominal injury of mechanical versus manual CPR following non-traumatic cardiac arrest

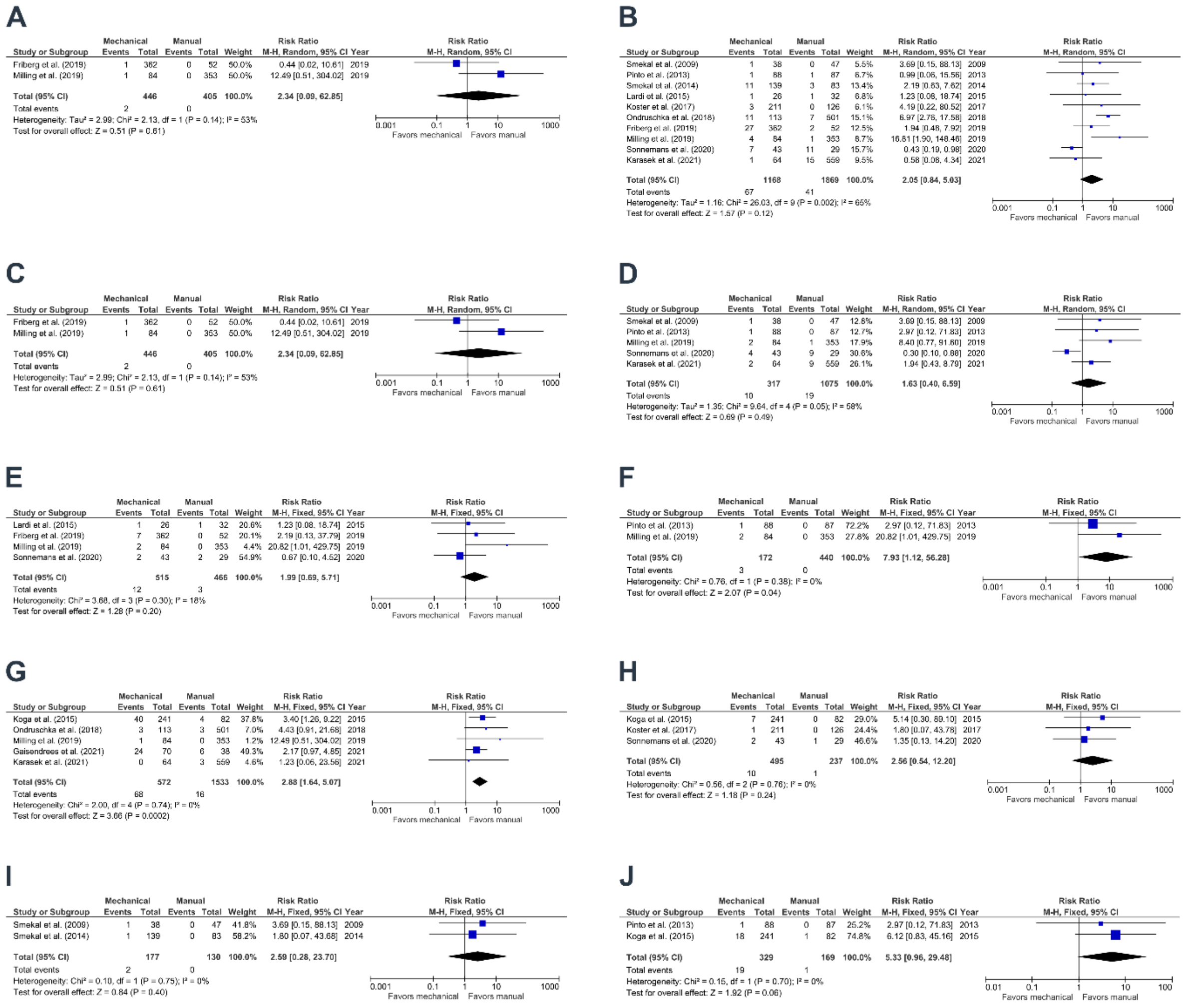

An intervention associated with less risk of CPR-related injury is favored.

A, stomach injury; B, liver injury; C, pancreas injury; D, spleen injury; E, kidney injury; F, bowel injury; G, hemoperitoneum; H, pneumoperitoneum; I, abdominal aorta injury; J, other abdominal injury.

CI, confidence interval; M-H, Mantel-Haenszel; SD, standard deviation.

Supplementary Online Materials 16: Funnel plots for any or thoracic CPR-related injuries sustained during manual versus mechanically assisted CPR

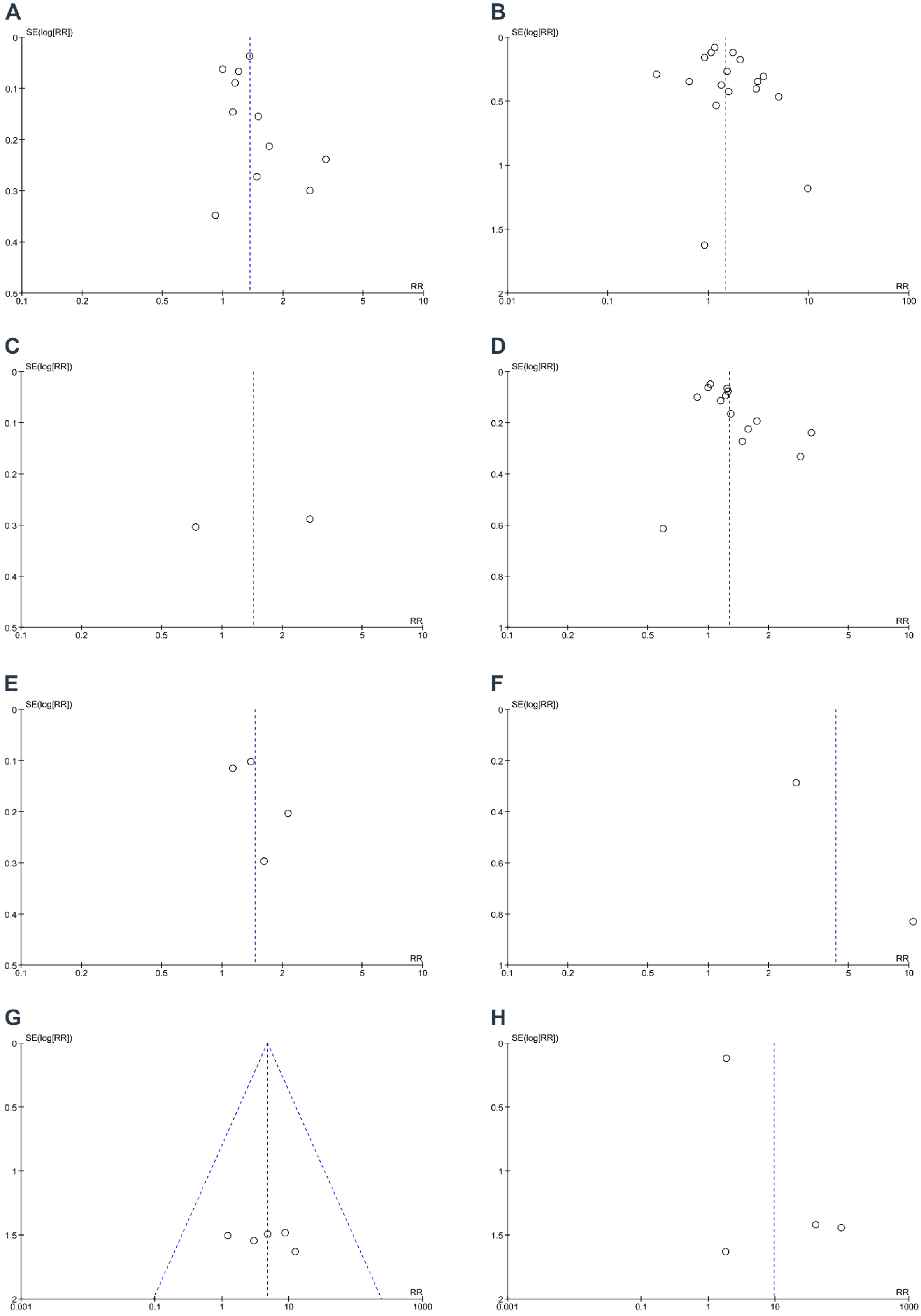

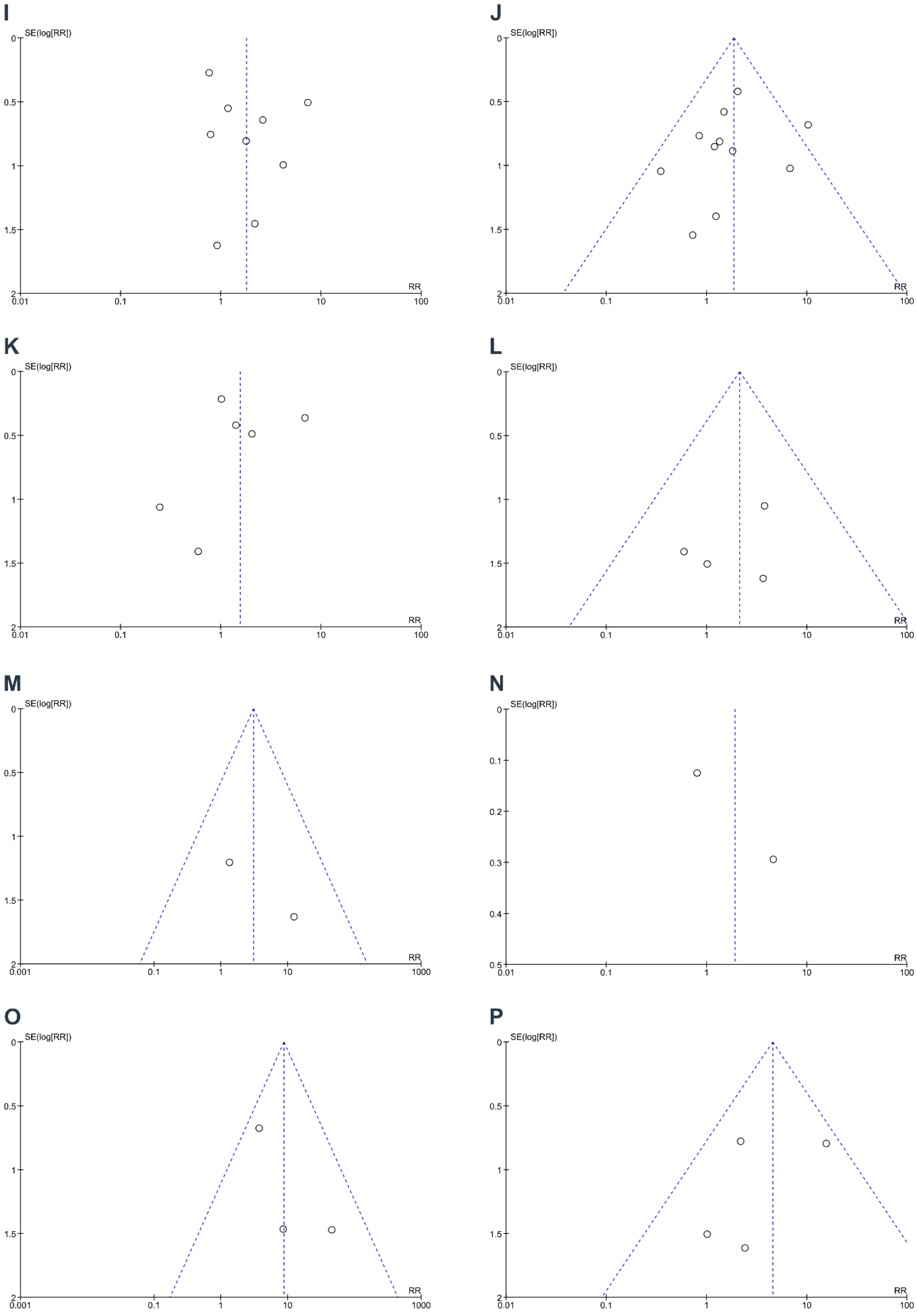
I
SE(log[RR])
RR
J
SE(log[RR])
RR
K
SE(log[RR])
RR
L
SE(log[RR])
RR
M
SE(log[RR])
RR
N
SE(log[RR])
RR
O
SE(log[RR])
RR
P
SE(log[RR])
RR

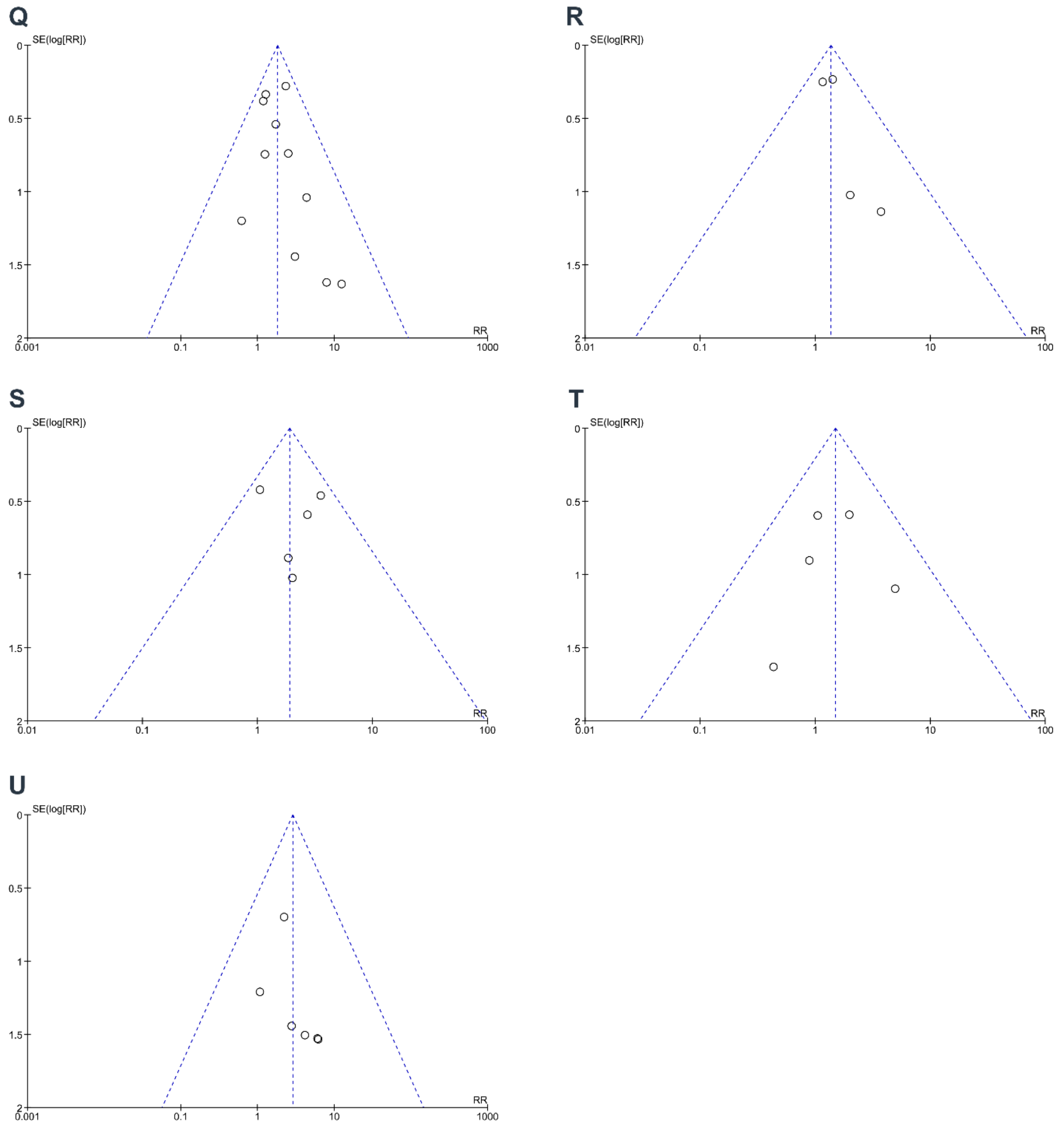

A, any CP-related injury; B, rib fracture(s); C, multiple rib fractures; D, flail chest; E, sternum fracture; F, vertebral fracture; G, extrathoracic chest wall injury; H, pneumothorax; I, hemothorax; J, pulmonary contusion; K, pulmonary hematoma; L, other pulmonary injury; M, retrosternal hematoma; N, hemomediastinum; O, pericardial or epicardial injury; P, myocardial contusion.

CPR, cardiopulmonary resuscitation.

Supplementary Online Materials 17: Funnel plots for abdominal CPR-related injuries sustained during manual versus mechanically assisted CPR

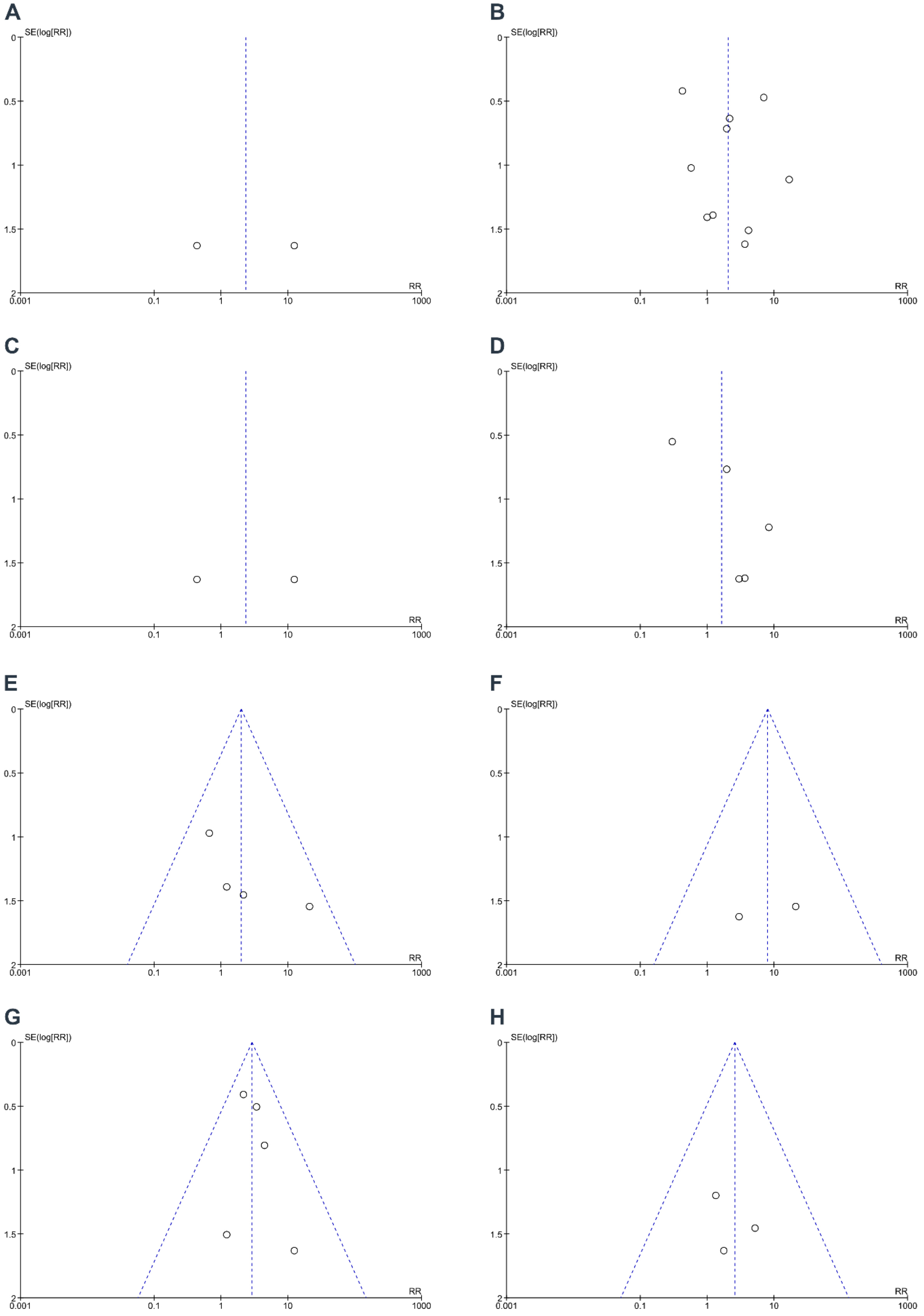

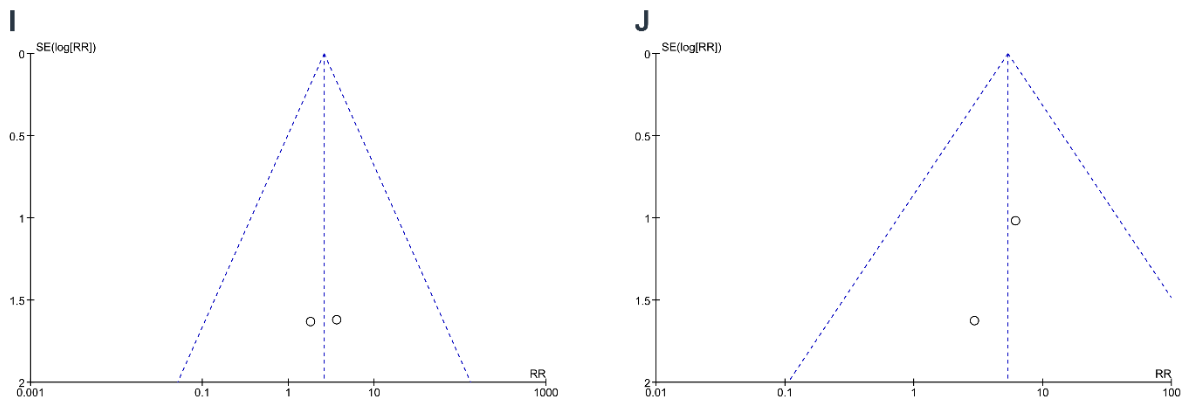

A, stomach injury; B, liver injury; C, pancreas injury; D, spleen injury; E, bowel injury; F, pneumoperitoneum; G, kidney injury; H, hemoperitoneum; I, abdominal aorta injury; J, other abdominal injury.

CPR, cardiopulmonary resuscitation.

Supplementary Online Materials 18: Forest plots detailing the risk ratio for CPR-related thoracic injury of manual versus mechanical CPR with a piston device following non-traumatic cardiac arrest

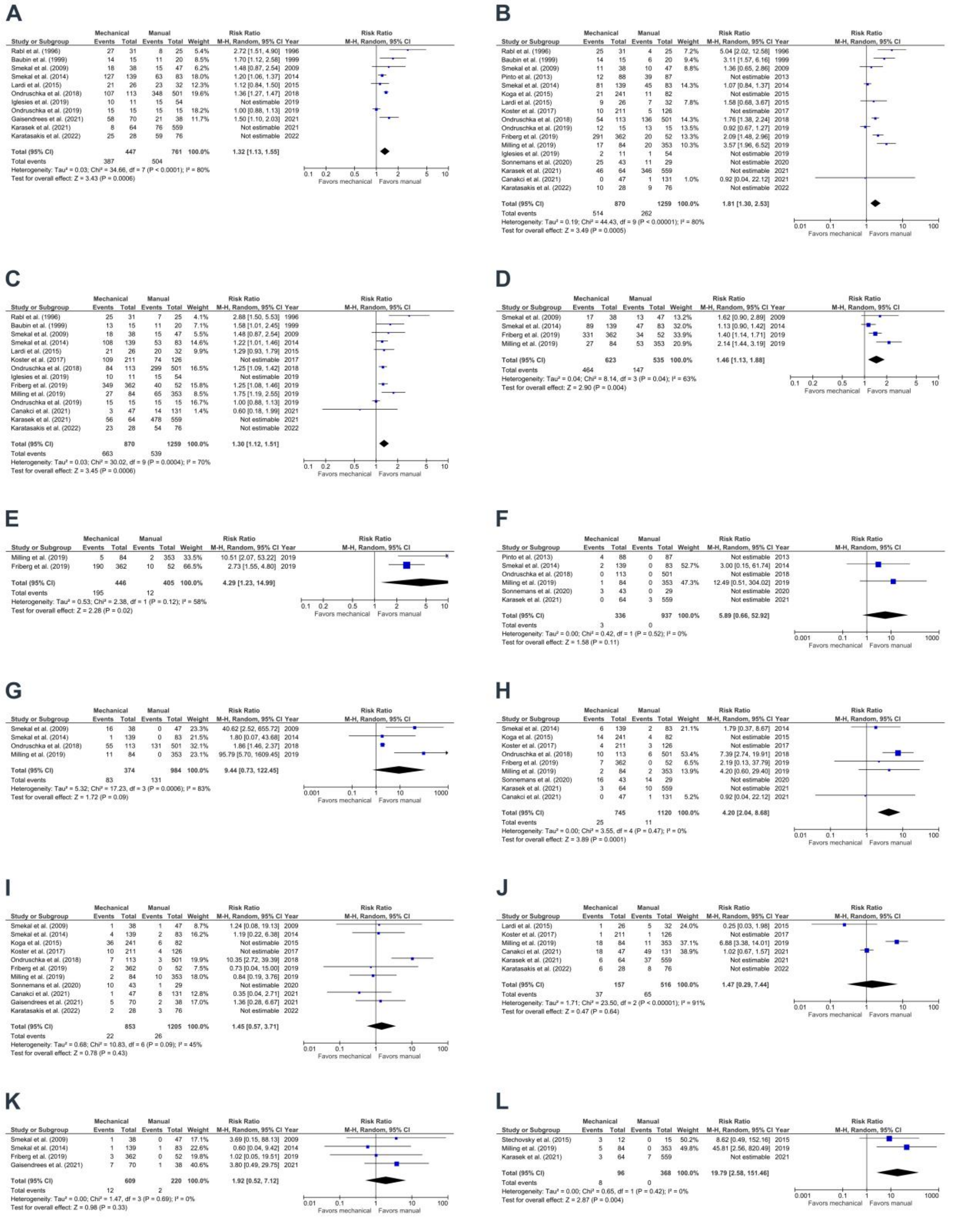

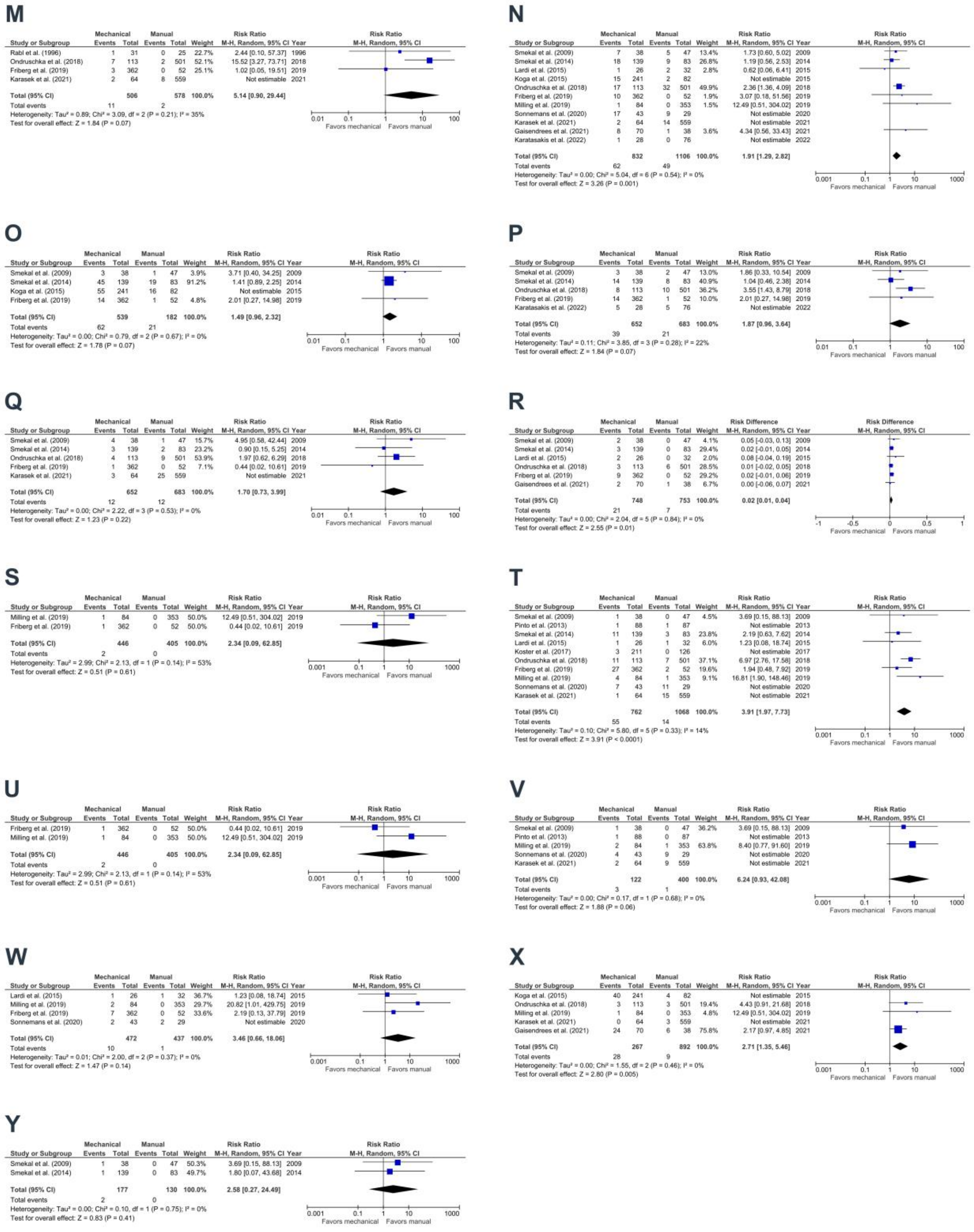

An intervention associated with less risk of CPR-related injury is favored.

A, any CPR-related injury; B, sternum fracture; C, rib fracture; D, multiple rib fractures; E, flail chest; F, vertebral fracture; G, extrathoracic chest wall injury; H, hemothorax; I, pneumothorax; J, pulmonary contusion; K, pulmonary hematoma; L, myocardial contusion;

M, cardiac laceration, rupture, or perforation; N, pericardial or epicardial injury; O, retrosternal hematoma; P, hemomediastinum; Q, other cardiac injury; R, thoracic vascular injury; S, stomach injury; T, liver injury; U, pancreas injury; V, spleen injury; W, kidney injury; X, hemoperitoneum; Y, abdominal aorta injury.

CI, confidence interval; M-H, Mantel-Haenszel; SD, standard deviation.

Supplementary Online Materials 19: Forest plots detailing the risk ratio for CPR-related thoracic injury of manual versus mechanical CPR with a load distributing band device following non-traumatic cardiac arrest

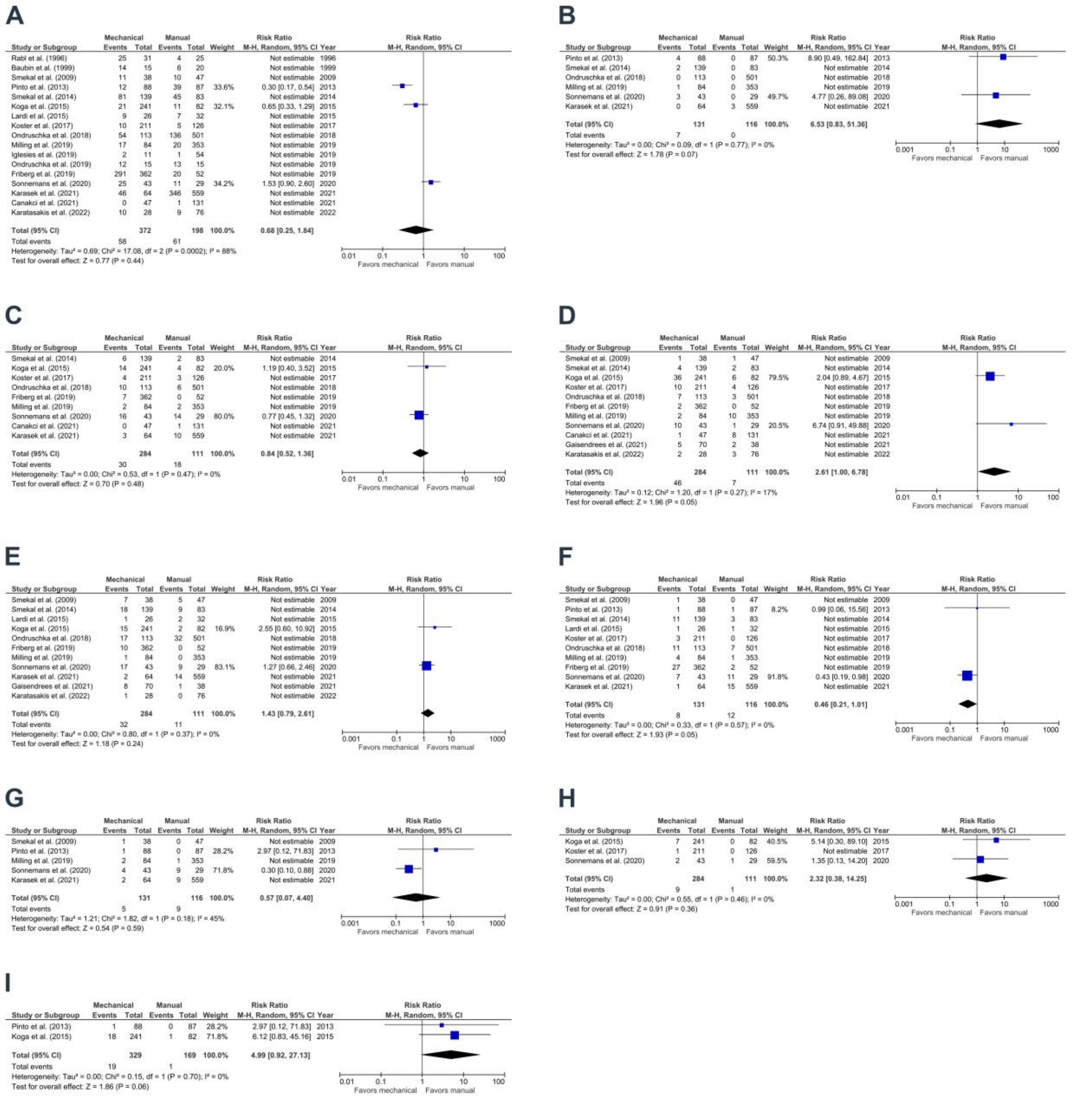

An intervention associated with less risk of CPR-related injury is favored.

A, sternum fracture; B, vertebral fracture; C, hemothorax; D, pneumothorax; E, pericardial or epicardial injury; F, liver injury; G, spleen injury; H, pneumoperitoneum; I, other abdominal injury.

CI, confidence interval; M-H, Mantel-Haenszel; SD, standard deviation.

Supplementary Online Materials 20: Funnel plots for CPR-related injuries sustained during manual versus mechanically assisted CPR with a piston device

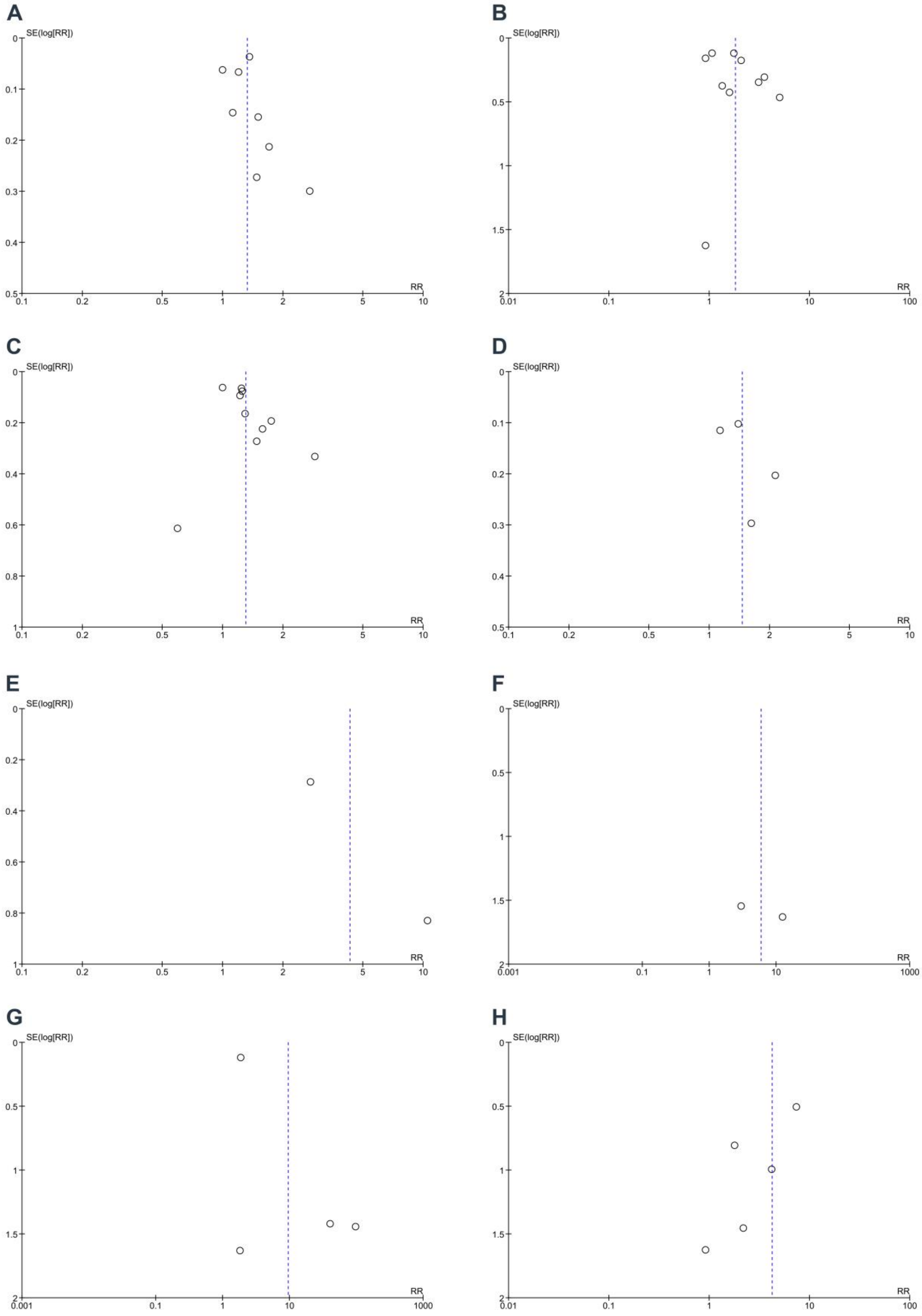

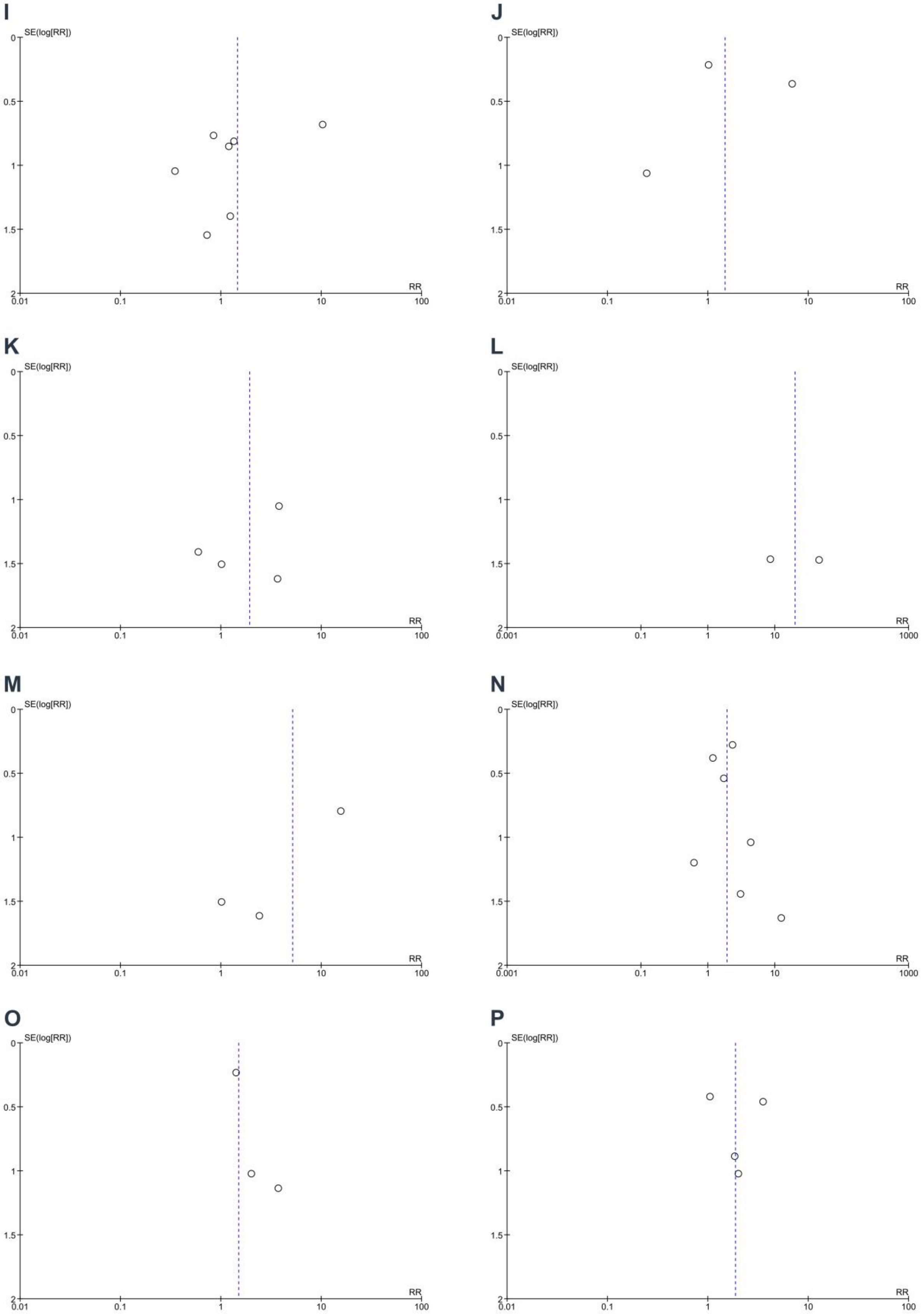
I
SE(log[RR])
RR
0.01
0.1
1
10
100
J
SE(log[RR])
RR
K
SE(log[RR])
RR
L
SE(log[RR])
RR
0.001
1000
M
SE(log[RR])
RR
N
SE(log[RR])
RR
O
SE(log[RR])
RR
P
SE(log[RR])
RR

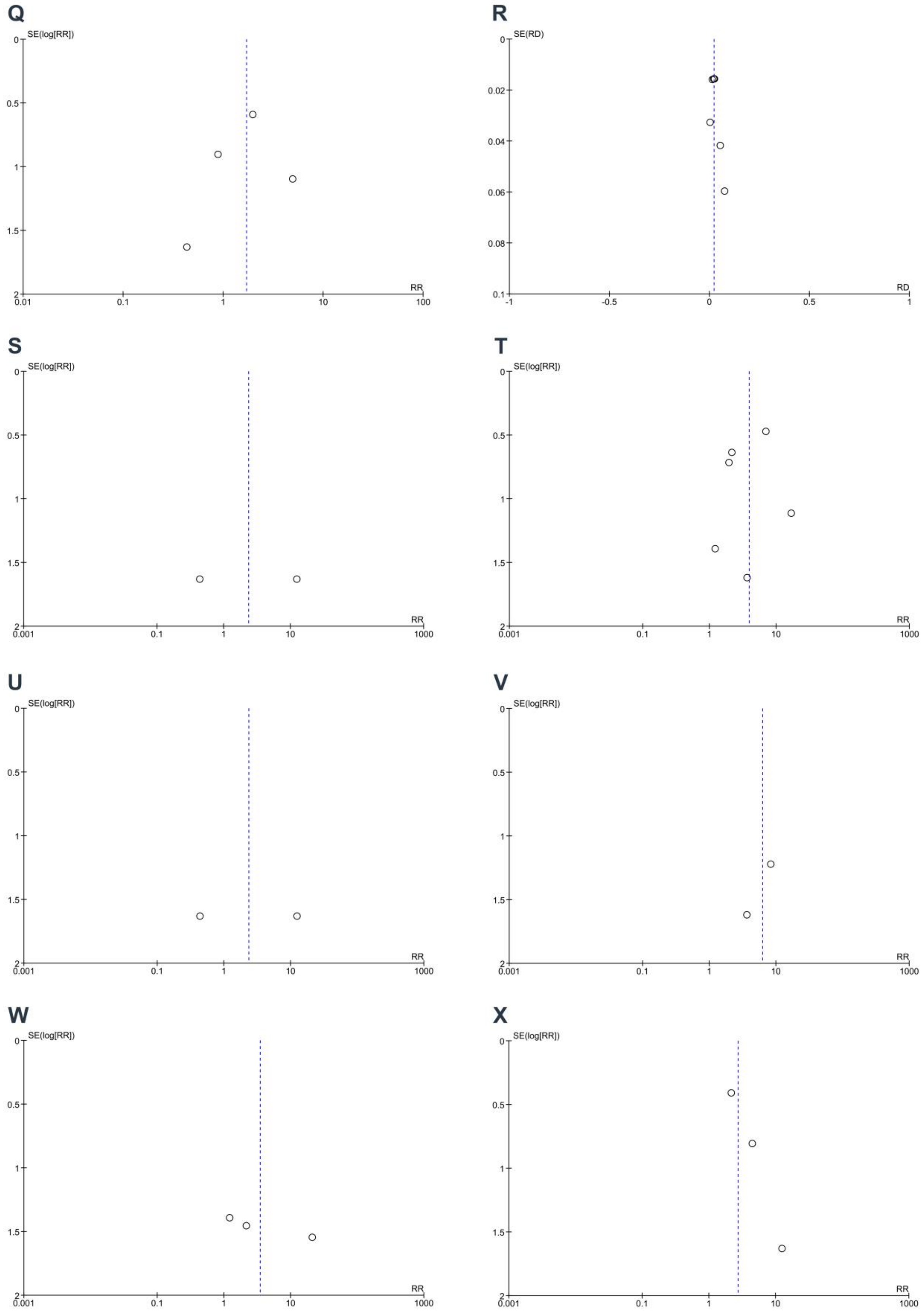
Q
SE(log[RR])
RR
R
SE(RD)
RD
S
SE(log[RR])
RR
T
SE(log[RR])
RR
U
SE(log[RR])
RR
V
SE(log[RR])
RR
W
SE(log[RR])
RR
X
SE(log[RR])
RR

Y

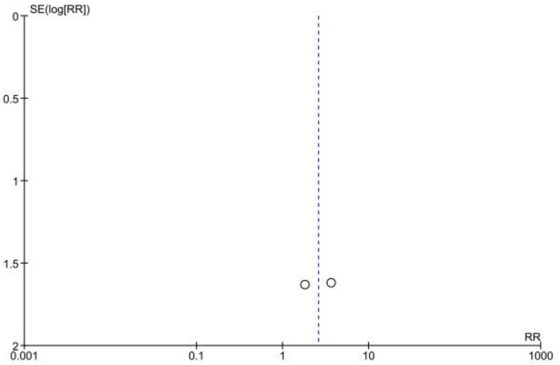

A, any CPR-related injury; B, sternum fracture; C, rib fracture; D, multiple rib fractures; E, flail chest; F, vertebral fracture; G, extrathoracic chest wall injury; H, hemothorax; I, pneumothorax; J, pulmonary contusion; K, pulmonary hematoma; L, myocardial contusion; M, cardiac laceration, rupture, or perforation; N, pericardial or epicardial injury; O, retrosternal hematoma; P, hemomediastinum; Q, other cardiac injury; R, thoracic vascular injury; S, stomach injury; T, liver injury; U, pancreas injury; V, spleen injury; W, kidney injury; X, hemoperitoneum; Y, abdominal aorta injury.

Supplementary Online Materials 21: Funnel plots for CPR-related injuries sustained during manual versus mechanically assisted CPR with a load distributing band device

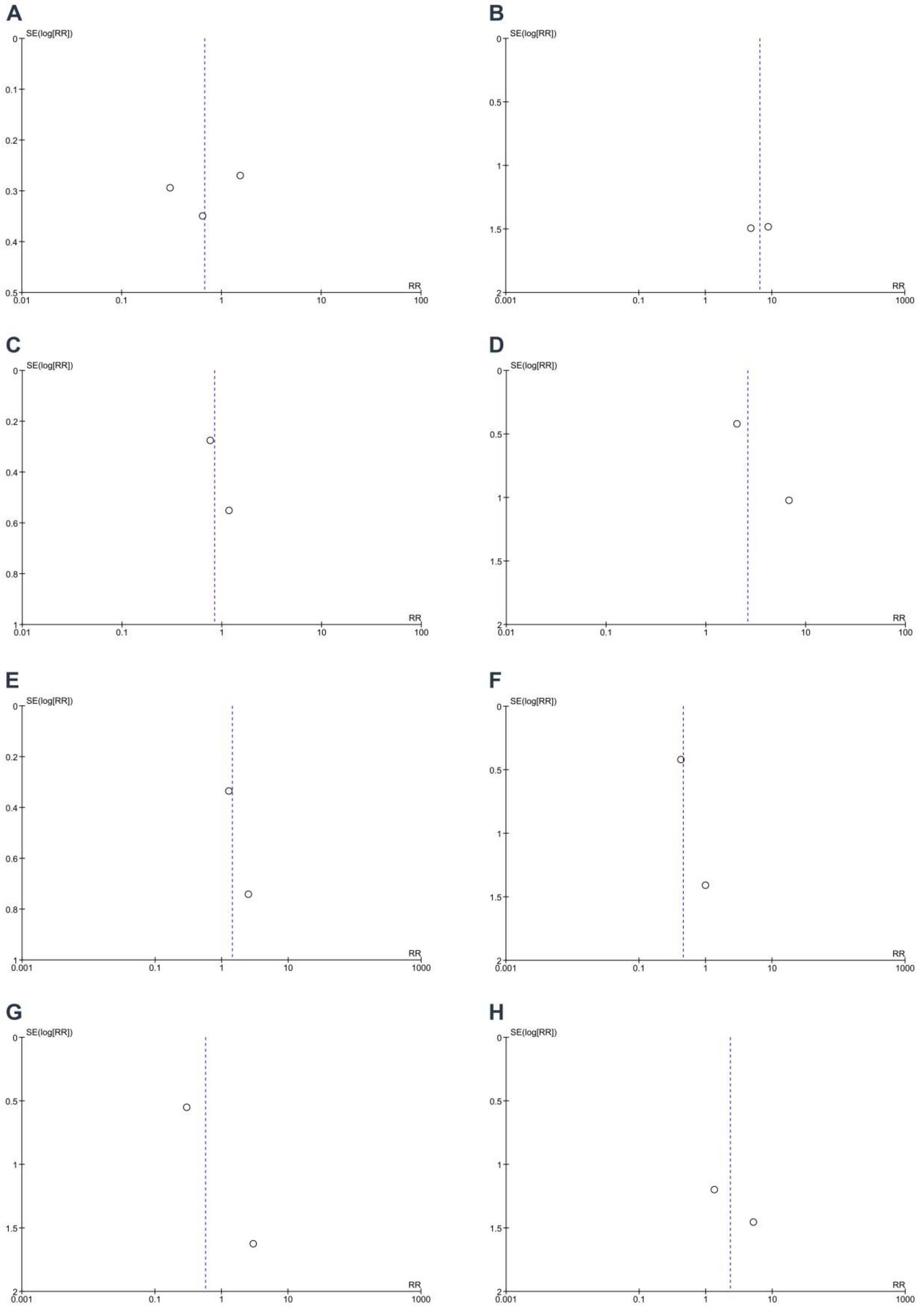

I

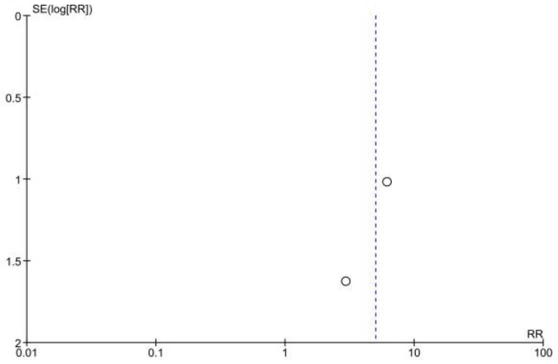

A, sternum fracture; B, vertebral fracture; C, hemothorax; D, pneumothorax; E, pericardial or epicardial injury; F, liver injury; G, spleen injury; H, pneumoperitoneum; I, other abdominal injury.
